# Crystallography of Extremophile Proteins—Structural Comparisons of Psychrophilic and Hyperthermophilic Rubredoxins

**DOI:** 10.3390/biom16050623

**Published:** 2026-04-22

**Authors:** Tzanko Doukov, Trenton F. Turpin, Dominic George, Caroline Cole, Kat Drumright, Madigan Rumley, Ryan Boyce, Francis E. Jenney, Stephen P. Cramer

**Affiliations:** 1 SLAC National Laboratory, SSRL, Menlo Park, CA 94025, USA; 2 University of Texas, Austin, TX 78712, USA; trentonturpin@utexas.edu (T.F.T.); mary@carolinecole.org (C.C.); 3 University of Saskatchewan, Saskatoon, SK S7N 5A2, Canada; dag488@mail.usask.ca; 4 University of California, Davis, CA 95616, USA; ksdrumright@ucdavis.edu; 5 University of Colorado-Boulder, Boulder, CO 80309, USA; madigan.rumley@gmail.com; 6 BIomedical Science, Philadelphia College of Osteopathic Medicine, Georgia Campus, Suwanee, GA 30024, USA; ryanbo2@pcom.edu (R.B.);; 7 SETI Institute, Mountain View, CA 94043, USA

**Keywords:** rubredoxin, extremophile, hyperthermophile, psychrophile, X-ray diffraction

## Abstract

Psychrophilic organisms are able to grow at temperatures down to −15 °C, while hyperthermophiles can multiply at temperatures up to 122 °C. What structural changes in extremophile proteins are needed to maintain stable and biochemically active structures under such conditions? Understanding how such extremophiles accomplish this is relevant for human health, biotechnology, and our search for life elsewhere in the universe. The purpose of the current study is to report and compare the structures of four rubredoxins (Rds), the first ever two experimental psychrophile bacteria structures (from Gram-positive *Clostridium psychrophilum* and Gram-negative *Polaromonas glacialis*) and two hyperthermophiles from the Gram-negative *Thermotoga maritima* bacterium and the archaeon *Pyrococcus yayanosii*, also a piezophile, as part of a program to understand structural variations that support both stability and function under extreme conditions. These structures were obtained using synchrotron radiation X-ray diffraction at 100 K. All four structures had the expected overall rubredoxin fold. Rubredoxin from the only aerobic psychrophilic bacterium *Polaromonas glacialis* had larger variations in sequence and structure, whereas the other psychrophilic bacterium showed properties closely related to hyperthermophile rubredoxins. Multi-subunit structures showed similar RMSD variability independent from their thermal adaptation status. We propose including functional information in the analysis since temperature optimization may not be the only determinant for a specific protein adaptation.

## 1. Introduction

For life to thrive under extreme conditions [1,2], its constituent proteins need to maintain stable and biochemically active structures [3,4,5,6]. Understanding the structure of extremophile proteins under such conditions is interesting in its own right, and it is also relevant for human health, biotechnology [7], and our search for life elsewhere in the universe [8]. The same is even true for proteins from ‘non-extremophiles’: how does structure change as a function of temperature and other conditions?

One approach to explaining extremophile protein stability is the ‘corresponding states’ proposal, which posits that proteins have evolved to achieve comparable flexibility at the optimal growth temperature for their respective organisms [9,10]. In support of this idea, neutron scattering experiments found comparable flexibility for psychrophiles and thermophiles at their respective adaptation temperatures (from 4 to 85 °C) [11]. However, some experiments conflict with its predictions. For example, using NMR-monitored amide hydrogen exchange experiments, hyperthermophilic *Pyrococcus furiosus* rubredoxin (*Pf* Rd) was found to have greater flexibility compared to the mesophile *Clostridium pasteurianum* (*Cpa* Rd) protein [12,13]. In a similar vein, from neutron scattering, NMR, and other measurements, hyperthermophilic P450 CYP119 was found to be more flexible than its mesophilic counterpart CYP101A at all temperatures above 200 K [14].

In a previous study using ^57^Fe measurements, we found comparable flexibility for hyperthermophilic *Pf* Rd compared to psychrophilic *Polaromonas glacialis* (*Pg*) Rd [15]. An additional surprise from our *Pf* Rd studies was the observation of a conformational change at around 343 K that produced modifications in hydrogen bonding [16]. However, it remains to be seen if *Pf* Rd represents a unique case, or if high temperature conformational changes are a general feature of extremophile proteins. As a starting point for addressing this issue, here we determined four new Rd structures from psychrophilic *Clostridium psychrophilum* (*Cpsy*) and *Pg*, as well as hyperthermophilic *Thermotoga maritima* (*Tm*) and hyperthermophilic and piezophilic *Pyrococcus yayanosii* (*Py*). Along with similar protein sequences (Table 1), we found comparable 100 K structures. These formed the basis for future studies at room temperature and ~373 K when possible.

## 2. Materials and Methods

### 2.1. Protein Preparation

The extremophile Rds were expressed in *Escherichia coli*. Recombinant vectors containing the genes that code for these rubredoxins were synthesized with codon optimization [26] for expression in *E. coli* in plasmid pET24d by GenScript (Piscataway, NJ, USA). We used *Cpsy* Rd (Genbank accession number WP_216289245.1), *Tm* Rd (Genbank accession number AKE30317.1), and *Py* Rd (Genbank accession number AEH24664.1). Production of *Pg* Rd (Genbank accession number WP_198026828.1) has been previously described [15].

The plasmids were electroporated into *E. coli* T7Express (New England Biolabs, Ipswich, MA, USA) and maintained in LB medium with 50 ug/mL kanamycin. The recombinant Rds were expressed and purified essentially as previously described [16] with a few modifications. *Py* Rd was purified the same way except that all three N-terminal forms (N-fMet, N-Met, and N-Ala (the native form)) were separated on hydroxyapatite chromatography. The *Tm* and *Cpsy* Rds were judged pure after gel filtration chromatography. Zinc forms of the recombinant proteins were prepared as described [16]. All protein solutions were concentrated to 40 mg/mL, 50 mM Tris pH 8.0, and 300 mM NaCl.

### 2.2. Protein Crystallization

All crystals were obtained with the hanging drop technique in Linbro plates with various NaCl concentrated solutions in the well. The specific conditions were: (a) Zn *Cpsy* Rd: Each drop contained 2 μL of protein solution, mixed with 2 μL 2.0 M NaKHPO_4_ and 1 μL *Cpsy* protein crystal derived seeds. The well contained 0.5 mL of 75% NaCl. (b) Fe *Pg* Rd: Each drop contained 2 μL of protein solution diluted to 14 mg/mL in 0.22 mM NAD, 50 mM Tris pH 8.0, 300 mM NaCl, and 2 μL of 2 M (NH_4_)_2_SO_4_ solution over 0.5 mL of a 70% saturated NaCl well solution. (c) Fe *Py* Rd: The coverslip contained a drop produced from 2 μL distilled water added to 2 μL protein solution, and 4 μL of 3.2 M NaKHPO_4_ and 100 mM Tris pH 7.0. Drops were equilibrated vs. a completely empty well. (d) Zn *Tm* Rd: Each drop contained 2 μL of protein solution, mixed with 2 μL 4.0 M (NH_4_)_2_SO_4_ and 1 μL *Tm* protein crystal derived seeds. The coverslip was equilibrated versus 0.5 mL of 80% saturated NaCl well solution.

### 2.3. Crystal Mounting

Protein crystals were harvested at the beamlines under ParatoneN oil, and the surface water layer was removed using a Hampton nylon loop (Hampton Research, Aliso Viejo, CA, USA). Finally, before mounting on the goniometer, the excess oil was dabbed away.

### 2.4. Diffraction Data Collection

All diffraction data were collected at SSRL. Data for a needle Zn *Cpsy* Rd crystal (1.2 × 0.15 × 0.10 mm) were collected using the helical data collection mode across the long side of the needle at BL12-2 at 0.7293 Å, allowing for a final data resolution of 0.84 Å in space group *P2_1_2_1_2_1_* (sg19) with a single Rd molecule per asymmetric unit. Despite the wavelength being far from the Zn edge (1.2836 Å), it was possible to phase *de novo* the structure with HKL2MAP [27]. A dataset at 1.7711 Å on the same crystal was used to confirm the presence of 3 K ions based on their anomalous signal.

An Fe *Pg* Rd crystal (150 × 100 × 50 μ) was used for data collection at 0.9795 Å at BL12-2 with 5% transmission, a 40 × 40 μ beam, and a calculated absorbed dose of 4.5 MGy. The strong anomalous signal from the Fe atoms allowed for *de novo* phasing with HKL2MAP [27] in space group *P4_3_2_1_2* (sg96), resulting in 7 unique Rd molecules in the asymmetric unit.

A single Fe *Py* Rd crystal (10 × 100 × 50 μ) dataset was collected at 0.9795 Å at BL12-2. Molecular replacement with an Fe *Pf* Rd model (1BRF) was used to locate the 3 subunits in the asymmetric unit of space group *P2_1_2_1_2_1_* (sg19). A weak anomalous signal from the Fe ions confirmed the metal center positions but was insufficient for *de novo* phasing.

A Zn *Tm* Rd crystal (150 × 150 × 100 μ) dataset was collected at 0.7749 Å at BL12-2 at a 1.02 Å data resolution in space group *P2_1_* (sg4). In addition, a second dataset from the same crystal at 1.2826 Å, corresponding to the Zinc peak wavelength from the MAD scan, was used for *de novo* phasing, resulting in 2 Rd molecules in the asymmetric unit.

### 2.5. Diffraction Data Processing

Diffraction data recorded on an Eiger 16M PAD detector (DECTRIS AG, Baden-Daettwil, Switzerland) [28] was processed with the XDS (19 January 2025) [29], Pointless (1.13.4) [29,30], Aimless (0.8.2) [31], CCP4 (9.0.008) [32], and STARANISO (2.4.16) programs, as implemented in the autoPROC software (1.0.5) [33].

### 2.6. Protein Sequence and Structure Analysis for Temperature Adaptation

Protein temperature adaptation, as well as all other properties, is encoded in the amino acid sequence. A pairwise sequence identities table was produced by BLASTp (2.17.0) [34], indicating the various degrees of difference for the novel and already known Rds in Table S1. Multisequence alignment was performed and a rooted phylogenetic tree was created using CLUSTAL Omega (1.2.4) [17] for Table 1 and Figure S1. Protein amino acid content was analyzed using ProtParam (EXpasy) [35,36]

Structural analysis was based on the output from the BANDIT web server for normalizing the B-factors per dataset [37]. Rigidity analysis was performed using the ProPHet rigidity web server [38]. Protein void volume and density calculations were produced using the ProteinVolume web server [39]. Surface charges were calculated in Pymol (3.1.4.1) [40]. New structures were analyzed using the ProteinTools server [41] and Amino Acid Interactions (INTAA) web server v2.0 [42].

## 3. Results

### 3.1. Overall Structures

**Figure 1:**
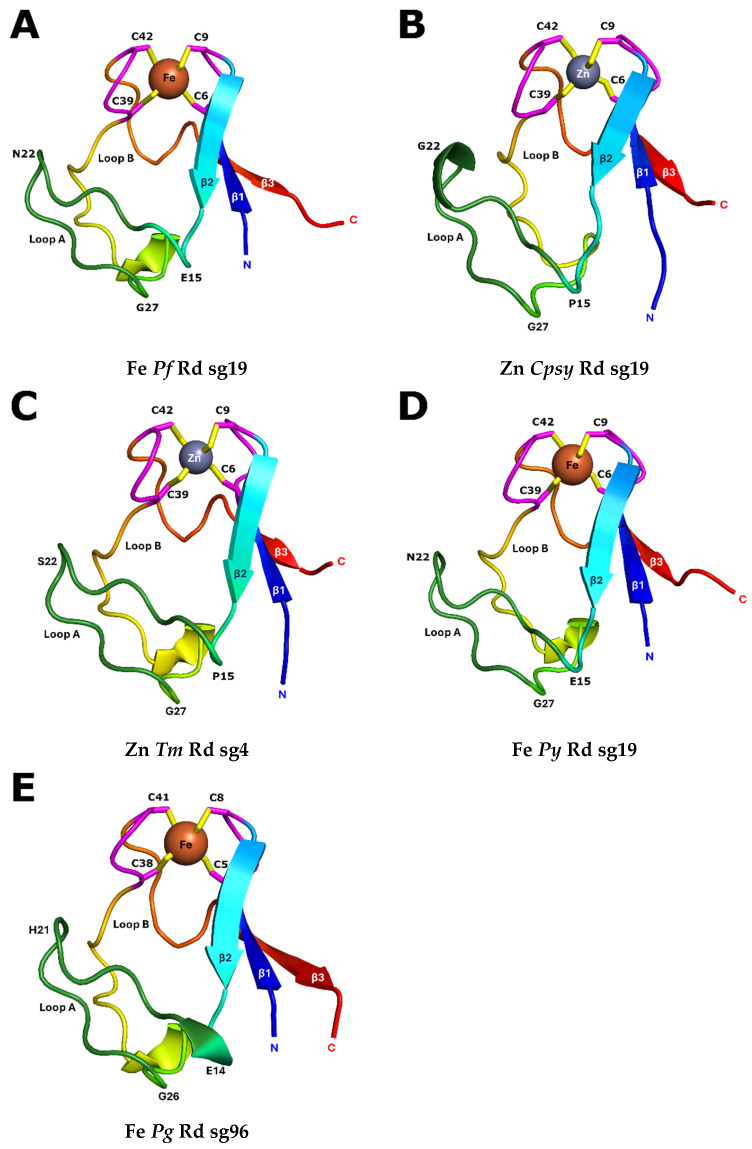
Rainbow cartoon representation for the structures for: (**A**) Fe *Pf* Rd, (**B**) Zn *Cpsy* Rd, (**C**) Zn *Tm* Rd, (**D**) Fe *Py* Rd, and (**E**) Fe *Pg* Rd. The figure was created with *Pymol* [40]. The label sg refers to the space group number.

The overall folds for these four new Rd structures were quite similar to the previous structure for *Pf* Rd [16], as illustrated in Figure 1. The structures all contain the essential features of the ‘consensus’ Rd, including the knuckles, *β*-sheets, aromatic cores, and loops A and B.

The Zn *Cpsy* Rd single monomer 0.84 Å structure was determined in space group *P2_1_2_1_2_1_* with R/R_free_ = 14.0%/15.8% and contained 71 waters and 3 K atoms. The Fe *Pg* Rd structure was determined in space group *P4_3_2_1_2* with seven independent Rd monomers to 1.83 Å, R/R_free_ = 17.2%/20.1%, and 227 identified waters and one Na ion. The Fe *Py* Rd structure with three independent Rd monomers was determined in space group *P2_1_2_1_2_1_* to 1.36 Å with R/R_free_ = 17.4%/21.2% and with 209 waters and a Na ion. The Zn *Tm* Rd structure with two independent Rd monomers was determined in space group *P2_1_* with R/R_free_ = 15.3%/18.1% and with 114 identified waters. The 100 K crystal structures for these four Rds have been submitted to the Protein Data Bank with the following PDB IDs: (a) Zn *Cpsy* Rd, 9ZDO; (b) Fe *Pg* Rd, 9ZDP; (c) Fe *Py* Rd, 9ZDH; and (d) Zn *Tm* Rd, 9ZDI. Experimental statistics for the crystallography experiments are listed in Table 2.

### 3.2. Metal Coordination

The metal coordination distances and angles confirmed the expected pseudo tetrahedral coordination by four cysteine residues with slightly longer bond lengths for the Zn-substituted proteins. Table S2 lists all the metal–ligand distances in the Rd molecules. Ligands shielded by aromatic residues Cys6 (by Phe49) and Cys39 (by Tyr11) have longer bonds to the metal center, but in some of the structures this general rule was not obeyed. It would be instructive to investigate with other spectroscopy methods if this is a real effect of part of ensemble sampling on crystal contacts within the multi-subunit crystals, or if it is due to lower crystal resolution.

### 3.3. Rd Fold Determinants on Molecular Level

The Rd fold is commonly described as a combination of the two knuckles around the iron and three antiparallel *β* strands. The structure is also held by additional specific bonds, which are also the most mechanically rigid parts of the molecules and the most resistant to H/D exchange. These include the already mentioned C6, C9, C39, and C42 coordinating the metal, the Y13 side chain with a T28 backbone, and the tight *β* turn backbone bond between K46 and F49 (*Pf* Rd numbering).

### 3.4. Asp-19 H-Bonding

**Figure 2:**
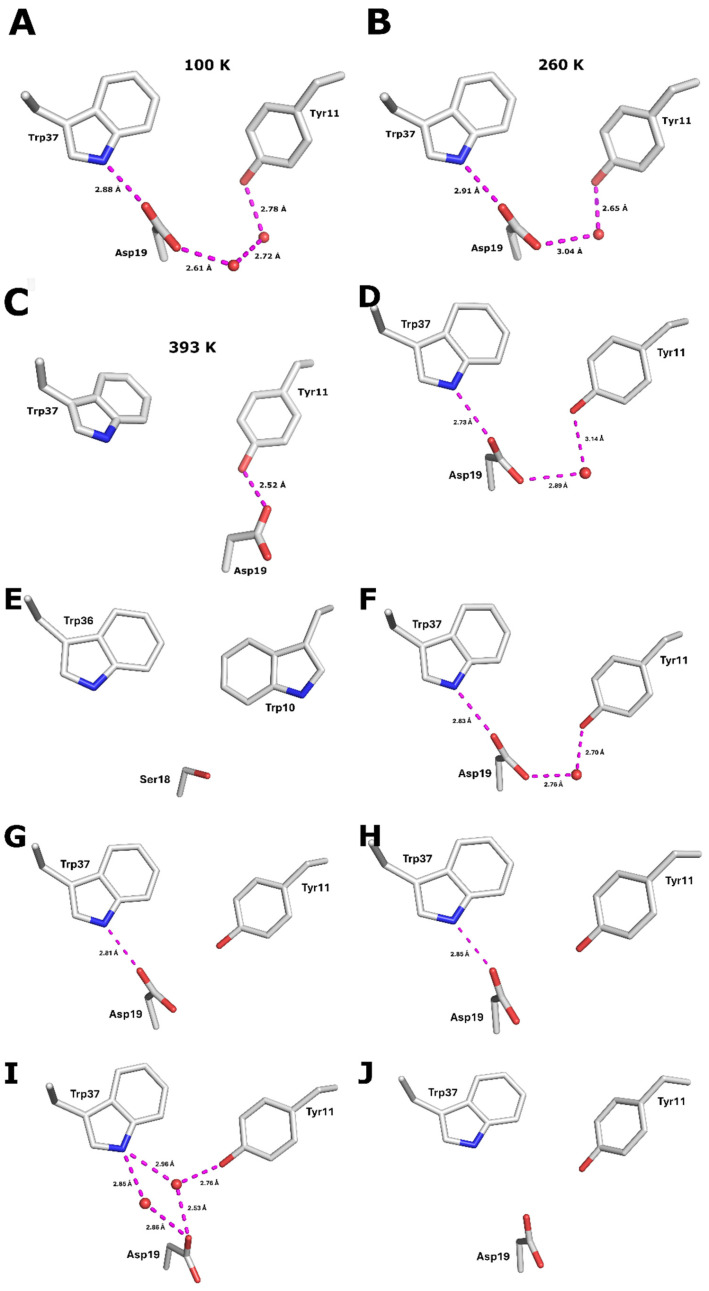
H-bonding patterns around Asp19: (**A**) *Pf* Rd at 100 K. (**B**) *Pf* Rd at 260 K. (**C**) *Pf* Rd at 393 K. (**D**) *Cpa* Rd at 100 K. (**E**) *Pg* Rd at 100 K. (**F**) *Cpsy* Rd at 100 K. (**G**) *Tm* Rd at 100 K. (**H**) *Py* Rd—subunit A at 100 K. (**I**) *Py* Rd—subunit B at 100 K. (**J**) *Py* Rd—subunit C at 100 K.

In our previous study of hyperthermophile *Pf* Rd [16], at 100 K we observed H-bonding from Asp19 to Trp37, with a connection to Tyr11 through two water molecules (Figure 2A). At 260 K there was only a single water bridge to Tyr11 (Figure 2B), and at 393 K Asp19 rotated to make a direct H-bond to Tyr11 (Figure 2C) [16]. For comparison, the mesophile *Cpa* Rd structure 1FHH at 100 K is analogous to the observed 260 K Zn *Pf* Rd structure with a single bridging water to Tyr11 (Figure 2D) [23].

The H-bonding pattern for Asp19 varies considerably in our new set of low-temperature Rd structures. In the psychrophilic *Cpsy* structure, the Asp19 H-bond network looks quite like 100 K *Pf* Rd, with a direct H-bond from Asp19 to Trp37 and a linkage to Tyr11 through two water molecules. It only lacks the stabilizing additional hydrogen bond from Asn22 in *Pf* Rd, since *Cpsy* Rd has Gly22 instead (Figure 2F). Both hyperthermophile structures have an Asp19 network conserved on a sequence level. The *Tm* structure also shows direct Asp19 to Trp37 H-bonding, but the connection to Tyr11 is more tenuous, with some water molecules at ~3.3 Å (Figure 2G). An interesting variability pattern is observed in *Py* Rd, where in subunit A the Asp19 keeps a similar hydrogen bond pattern to Trp37 (Figure 2H), while in subunit B it utilizes a bridging water molecule to simultaneously reach the Trp37 ring as well as the Tyr11 ring (Figure 2I). Surprisingly, the Asp19 is ~4.6 Å and ~3.9 Å away from both Trp37 and Tyr11 in subunit C without a well-ordered density in any bridging positions (Figure 2J). Note that corresponding amino acids in the *Pf* and *Py* sequences occupy three different spatial positions depending on the subunit in the unit cell (Figure 2A,H–J). Finally, in *Pg* Rd, there is an Ser18 (−1 shift for all residues compared to the rest of the sequences) in the place of Asp19, and a Trp10 in the place of Tyr11, which lacks the hydrogen bond network (Table 1) (Figure 2E).

### 3.5. Glu15 H-Bonding

**Figure 3:**
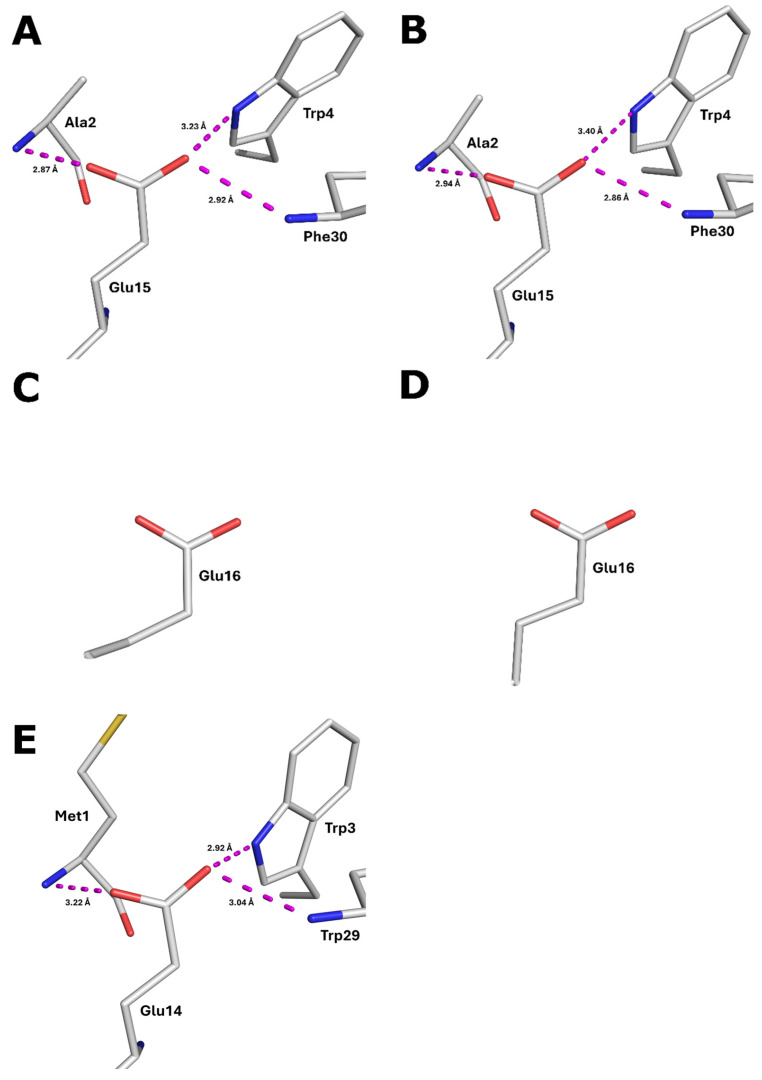
H-bonding patterns around Glu15 at 100 K. (**A**) *Pf* Rd (100 K). (**B**) *Py* Rd. (**C**) *Cpsy* Rd. (**D**) *Tm* Rd. (**E**) *Pg* Rd.

Extensive H-bonding around Glu15 has often been invoked as a source of stability for the hyperthermophile *Pf* Rd [43,44,45]. In all of the *Pf* Rd structures, Glu15 interacts with the indole ring of Trp4 through a long hydrogen bond and the amide O of Phe30 and the N-terminal Ala2 (Figure 3A). In *Py* Rd (Figure 3B) the Glu15 hydrogen bonding pattern is conserved. In contrast, in psychrophile *Cpsy* Rd (C) and hyperthermophile *Tm* Rd (D), as well as in the mesophile *Cpa* Rd, there is a Pro at position 15, disrupting the hydrogen bonding network. Finally, in *Pg* Rd, the analogous residues are Glu14, and Trp3 and Met1 (Arg5 H-bonds in ZnTmRd—Figure 3E), but the H-bonds to the N-terminal residue are too long or in an unfavorable geometry in four (subunits B, E, F, and G) of the seven subunits, making the H-bonding network requirement very flexible/complex depending on the structural content inside the crystal.

### 3.6. Arg5 -Glu50 Salt Bridge in ZnTmRd

**Figure 4:**
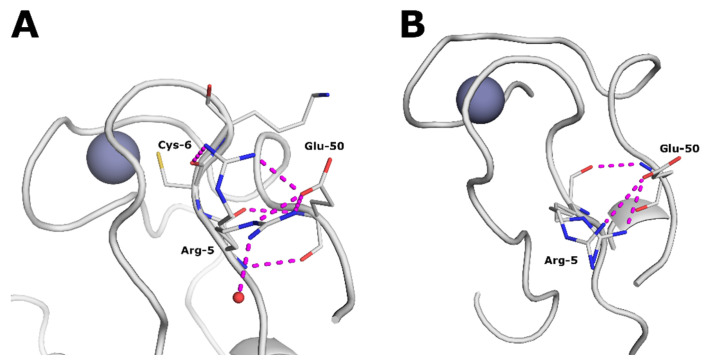
H-bonding patterns around Arg5 at 100 K in Zn *Tm* Rd structure. (**A**) Subunit A. (**B**) Subunit B.

*Tm* Rd lost the favorable and energy stabilizing Glu15 hydrogen bond network but remained a hyperthermophile. How could that be possible? Careful examination of the amino acids’ interaction energies using the INVAA server showed that a new favorable salt bridge was formed between Arg5 and Glu50. It was present in both subunits of the Zn *Tm* Rd structure as illustrated in Figure 4.

### 3.7. Water Molecules

The number of water molecules observed per protein molecule is included in Table S3. We found this mostly reflects the resolution for each structure, rather than the differential hydration of the proteins.

### 3.8. Sequence and Structure Analysis of Temperature Adaptation

A general theory of cold adaptation is that increased molecular flexibility and decreased stability result in higher enzymatic activity [9,46]. Analyzing electron transfer psychrophilic enzymes could be very complex and challenging. This complex, multifactorial adaptation process involves fitting different functional partners to accommodate aerobic or anaerobic species and varying pressure requirements, whereas the electron transfer process itself does not rely on major structural changes. [15].

Sequences of the novel bacterial and archaeal rubredoxins were compared with previously determined rubredoxin structures (Table 1). A phylogenetic tree of that comparison (Figure S1) indicates *Polaromonas glacialis* rubredoxin belongs to the aerobic branch, suggesting a different functional role in aerobic metabolism. Based on multiple protein families with psychrophilic, mesophilic and thermophilic X-ray structure representatives, Feller summarized the psychrophilic protein adaptations as: (a) an increase in glycine residues favoring higher flexibility, (b) a decrease in proline in loop regions, which usually are rigidifying protein structures, (c) a decrease in arginine residues which also rigidify structures by salt bridges and/or hydrogen bonds, and (d) a reduction in the size of the amino acids in the hydrophobic core, therefore weakening the stabilizing hydrophobic forces [47]. Additionally, the role of increased negative surface charge in enabling flexibility at low temperatures was suggested [47]. Data based on these criteria is listed in Table 3. Psychrophilic *Cpsy* and *Pg* rubredoxins have unexpectedly small negative surface charges.

“Determination of molecular flexibility is complex as it requires the definition of the types and amplitudes of atomic motions as well as a timescale for these motions.” [47]. Preliminary circular dichroism spectra for the psychrophile *Pg* Rd indicate melting temperatures (>50 °C) at the *β*-sheet wavelengths in the thermophilic temperature range (manuscript in preparation). On a structural level, the very wide RMSD subunit distribution (Table S4), the high average B-factor (~47, Table 2), and the B-factor distribution (Figures S3–S5) for the *Pg* subunits indicate intrinsic flexibility. Secondary structure assignment by the STRIDE algorithm [48] also showed variations in the overall very conserved fold (Table S5). Even in thermophilic rubredoxins (Figures S2–S4) with multiple subunits, variations were observed in flexible loop B, suggesting that some of the structural information may be content-dependent on crystal contacts, solvent content differences, and/or precipitant concentration and nature (inorganic salt or PEG).

Local structural rigidity could be identified computationally with the ProPHet rigidity server. While the thermophilic proteins tend to have higher absolute peaks (>120 for Y13), one of the psychrophiles, *Cpsy* Rd, with a low solvent content also has a similar peak height, suggestive of content-dependent modulation of the properties (Figure S5).

Another way to destabilize the proteins is having larger cavities and voids in the psychrophile proteins. *Cpsy* Rd and some of the *Pg* Rd subunits have lower protein densities, but some of the *Pg* subunits have densities higher than mesophile or thermophile proteins in the table (Table S6). Therefore, using that criterion to identify temperature adaptation is not advisable. *Pab* Rd data illustrates that protein density is lower at room temperature than at 100 K. The *Pf* Rd crystal (PDB 5NW3) exhibits the highest protein density, owing to its lower solvent content and higher resolution.

The amino acid interactions server (INTAA) derives interaction energies between amino acids. Its concept was verified by an ab initio study of rubredoxin [49]. The current version of the server incorporates both detailed interaction energy calculations and amino acid conservation within an interaction energy matrix (IEM). A convenient way to evaluate the overall state of the protein is a Scatter Plot of the interaction energy versus information content (IC) as a measure of amino acid conservation within multisequence alignments. It brought to our attention the strong stabilizing effect of Arg5 in *Tm* Rd, an amino acid not conserved universally in rubredoxins. Total IE-IC plots are included in Figure S6.

## 4. Discussion

In this work we presented a set of four new Rd crystal structures—two from hyperthermophiles and, for the first time, two from psychrophiles. All four structures follow the conserved Rd fold and metal center coordination, and they all include a six-aromatic-residue core. However, there are some interesting differences.

On a sequence level, the Rd from the psychrophilic *Pg* bacterium (the only aerobe in the set) differs significantly from the other three with the lack of D19 (here S18) H-bond networks, as well as substitutions within the core aromatics (W10 instead of Y11 and W29 instead of F30) (Table S1). Its second amino acid in the Fe binding motif CXXC is E40, compared to the most common L41 (*Cpa*, *Cpsy*, *Py*, and *Tm*) or I41 (*Pf*). We speculate that the charged carboxylate residue from the glutamate could lead to a different redox potential for *Pg*, compared to the anaerobic organisms. Supporting this idea, other Rds from aerobes have a D at position 41, and the charged carboxylate from the aspartate should have a similar effect. When compared to known Rd sequences (Table 1), *Pg* Rd fits into the RubB (Rd type 2) family which includes *Mt* Rd [20] and *Pa* Rd [21], both of which are involved in dioxygen chemistry with Cytochrome P450 or Alkane monooxygenase (AlkB). Although the redox partners of *Pg* Rd have not been assigned, both P450 and AlkB are present and seem likely candidates.

The other psychrophilic protein, *Cpsy* Rd, contains a rare seventh aromatic amino acid. Phe47 is located on a solvent-exposed loop, where it hinders stabilizing hydration of the protein. Although aromatic residues strongly enhance stability, Phe47’s contribution is the smallest of all *Cpsy* aromatic residues. Its B-factors are roughly three times higher than the B-factors of the buried aromatic residues. The closest aromatic residue is symmetry-related Tyr11 at ~4.9 Å. *Cpsy* Rd has Pro15 instead of the stabilizing hydrogen-bonded Glu15 found in *Pf* rubredoxin. This is reflected in the observation of multiple conformations for the aromatic residue at position 30 (Phe30), which is usually very ordered in other rubredoxins (e.g., Trp29 in *Pg* Rd).

Organisms utilize multiple molecular mechanisms to adapt to cold environments [50], increasing metabolic activity despite reaction rates that are unfavorable according to the Arrhenius Law. It is proposed that the general mechanism involves increasing flexibility by disrupting stabilizing interactions. Such molecular changes lead to decreased protein stability [51]. There is not a universal molecular mechanism for cold adaptation. Adaptations vary between different protein groups. Because rubredoxin is a very small electron transfer protein, its molecular adaptation signals are subtle, making it difficult to differentiate between psychrophiles and thermophiles. From our analysis we found that only decreased negative surface charge correlates with expected lower thermal stability in rubredoxins (Table 3). Rubredoxins exhibit a melting temperature significantly higher than the optimal growth temperature of the organism, presenting an unexpectedly large discrepancy. Surprisingly, both psychrophilic and thermophilic proteins showed significant structural variations across multiple crystal subunits. Instead of acting as solid, uniform units, thermophilic proteins displayed varied B-factor profiles.

## 5. Conclusions

We reported X-ray diffraction rubredoxin structures from the anaerobic hyperthermophile bacterium *Thermotoga maritima* and piezophile archaeon *Pyrococcus yayanosii*. We also reported for the first time the experimental structures of two psychrophilic rubredoxins—from anaerobe *Clostridium psychrophilum* and aerobe *Polaromonas glacialis* bacteria. Their overall structures were remarkably preserved within the rubredoxin fold with three antiparallel *β*-strands and four cysteine–iron binding sites. We applied multiple protein sequence analyses and structure-based tests to identify the likely origin of temperature adaptation. Aside from an unexpected reduction in calculated surface charge in both psychrophiles, no clear molecular clues explained the adaptation from psychrophiles to mesophiles and hyperthermophiles.

While analyzing crystals with multiple subunits, we stumbled across the significant role that crystal contacts, solvent content, data collection temperature, and resolution play upon structural malleability. This affected not only the supposedly more flexible psychrophiles but also the supposedly more rigid thermophiles. Performing future experiments at physiological temperature and pressure conditions would allow for a better capture of molecular signatures related to temperature adaptation.

## Figures and Tables

**Table 1:** Sequences for rubredoxins with structures solved in this paper or previously aligned by *CLUSTAL Omega* [17]. ^1^ *Pseudomonas oleovorans* 2Fe Rd C-terminal starting at residue 119 [18,19], ^2^ *Mycobacterium tuberculosis* Rd 2 [20], ^3^ *Pseudomonas aeruginosa* Rd [21], ^4^ *Clostridium pasteurianum* Rd [22,23], ^5^ *Pyrococcus abyssi* Rd [24], and ^6^ *Pyrococcus furiosus* Rd. Growth temperatures are taken from the BacDive database [25]. Conserved cysteines—**C**, prolines—**P**, other prolines—**P**, and core aromatics—**W**, **Y**, and **F**. The rooted phylogenetic tree of the alignment is included in Supplemental Figure S1.

Organism	Sequence	Growth Temp. °C	PDB ID
*Cpsy*	MNK ** Y ** V ** C ** LV ** C ** G ** Y ** E ** Y ** D ** P ** EIGDLEGGIK ** P ** GTK ** F ** EDL ** P ** ED ** W ** L ** C P ** L ** C ** GVTK ** F ** D ** F ** EKI	4	9ZDO
*Pg*	**–** MT ** W ** M ** C ** LI ** C ** G ** W ** I ** Y ** D **E** ALG **SP** EHGIAAGT ** P W ** SQV ** P ** MN ** W ** T **CPEC** GARKED ** F ** EMVQM	10	9ZDP
*Po * ct ^1^	YLK ** W ** I ** C ** IT ** C ** G **H** I ** Y ** D **E** ALGDEAEGFT ** P ** GTR ** F ** EDI ** P ** DD ** W ** C **CPDC** GATKED ** Y ** VLYEEK	30	1A24
*Mt 2 * ^2^	YKL ** F ** R ** C ** IQ ** C ** G ** F ** E ** Y ** D **E** ALG ** W P ** EDGIA **A** GTR ** W ** DDI ** P ** DD ** W ** S **CPDC** GAAKSD ** F ** EMVEVARS	37	7A9A
*Pa * ^3^	MKK ** W ** Q ** C ** VV ** C ** G **L** I ** Y ** D **E** AKG ** W P ** EEGIE **A** GTR ** W ** EDV ** P ** ED ** W ** L **CPDC** GVGKLD ** F ** EMIEIG	37	2V3B
*Cpa* ^4^	MKK ** Y ** T ** C ** TV ** C ** G ** Y ** I ** Y ** N ** P ** EDGD ** P ** DNGVN ** P ** GTD ** F ** KDI ** P ** DD ** W ** V ** C P ** L ** C ** GVGKDQ ** F ** EEVEE	37	1FHH
*Tm*	MKK ** Y ** R ** C ** KL ** C ** G ** Y ** I ** Y ** D ** P ** EQGD ** P ** DSGIE ** P ** GT ** P F ** EDL ** P ** DD ** W ** V ** C P ** L ** C ** GASKED ** F ** E ** P ** VE	80	9ZDI
*Pab * ^5^	MAK ** W ** R ** C ** KI ** C ** G ** Y ** I ** Y ** D **E** DEGD ** P ** DNGISPGTK ** F ** EDL ** P ** DD ** W ** V ** C P ** L ** C ** GA ** P ** KSE ** F ** ERIE	95	1YK5
*Py*	MAK ** W ** R ** C ** TV ** C ** G ** Y ** I ** Y ** D **E** EEGD ** P ** DNGVL ** P ** GTK ** F ** EEL ** P ** DD ** W ** V ** C P ** L ** C ** GA ** P ** KDM ** F ** EKVD	98	9ZDH
*Pf * ^6^	MAK ** W ** V ** C ** KI ** C ** G ** Y ** I ** Y ** D **E** DAGD ** P ** DNGIS ** P ** GTK ** F ** EEL ** P ** DD ** W ** V **CPIC** GA ** P ** KSE ** F ** EKLED	100	5NW3
*#*	000000000111111111122222222223333333333444444444455555		
*#*	123456789012345678901234567890123456789012345678901234		

Yellow boxes highlight non-conserved Prolines, while cyan boxes highlight partially conserved Asp-19 and Leu-41.

**Table 2:** Data collection, processing, and refinement statistics for the four new Rd crystal structures at 100 K.

Protein	Zn *Cpsy* Rd	Fe *Pg* Rd	Fe *Py* Rd	Zn *Tm* Rd
PDB ID	9ZDO	9ZDP	9ZDH	9ZDI
Temperature (K)	100	100	100	100
SSRL beamline	BL12-2	BL12-2	BL12-2	BL12-2
Crystal size (mm)	1.20, 0.15, 0.10	0.15, 0.10, 0.05	0.01, 0.10, 0.05	0.15, 0.15, 0.10
Wavelength (Å)	0.72929	0.97946	0.97946	0.77488
Resolution range (Å)	27.39–0.84 (0.95–0.84)	69.29–1.83 (2.04–1.83)	33.25–1.36 (1.50–1.36)	25.26–1.02 (1.08–1.02)
Space group	*P2_1_2_1_2_1_*	*P4_3_2_1_2*	*P2_1_2_1_2_1_*	*P2_1_*
Unit cell: a, b, c (Å) α β γ	27.48, 37.02, 40.72 90 90 90	73.90, 73.90, 199.35 90 90 90	26.91, 63.51, 78.06 90 90 90	27.02, 50.03, 33.93, 90 110.8 90
Total reflections	231,110 (9614)	1,951,770 (86,408)	175,948 (8462)	209,291 (10427)
Unique reflections	25,522 (1276)	36,053 (1803)	17,219 (861)	34,060 (1703)
Multiplicity	9.1 (7.5)	54.1 (47.9)	10.2 (9.8)	6.1 (6.1)
Completeness (spherical) (%)	66.5 (10.9)	73.0 (13.5)	57.8 (11.4)	79.1 (25.6)
Completeness (ellipsoidal) (%)	91.0 (50.6)	95.3 (69.9)	89.7 (57.5)	88.0 (50.5)
Mean I/sigma(I)	9.3 (1.9)	14.1 (1.6)	6.4 (1.5)	6.2 (1.8)
Wilson B-factor (Å^2^)	11.941	20.709	20.306	14.477
R-merge	0.115 (1.56)	0.277 (6.053)	0.433 (3.708)	0.205 (3.317)
R-pim	0.040 (0.838)	0.038 (0.862)	0.138 (1.216)	0.089 (1.427)
CC_1/2_	0.997 (0.515)	0.998 (0.608)	0.979 (0.837)	0.985 (0.340)
ISa	17.97	14.82	14.0	9.99
Mosaicity	0.51	0.11	0.14	0.16
Refinement				
Refinement range	27.39–0.84 (0.95–0.84)	69.29–1.83 (1.88–1.83)	33.25–1.36 (1.39–1.36)	25.26–1.02 (1.04–1.02)
R_work_ (%)	14.0 (31.6)	17.2 (29.8)	17.4 (27.6)	15.3 (26.6)
R_free_ (%)	15.8 (0.00)	20.1 (51.3)	21.2 (29.7)	18.1 (31.8)
No. of non-H atoms				
Total	537	3153	1480	970
Macromolecules	466	2918	1257	854
Ligands (Zn/Fe, Na/K)	1 Zn, 3 K	7 Fe, 1 Na	3 Fe, 1 Na	2 Zn
Water	71	227	209	114
R.m.s. deviations				
Bond lengths (Å)	0.012	0.007	0.008	0.011
Angles (°)	2.240	1.704	1.589	1.953
Average B-factor (Å^2^)	17.134	47.743	17.478	13.718
Macromolecules	15.841	47.778	15.915	12.652
Ligands (Zn/Fe, Na/K)	4.21 Zn, 18.78 K (3)	39.8 Fe(7), 34.0 Na	9.33 Fe(3), 26.3 Na (1)	8.1 Zn (2)
Water	26.104	47.293	26.931	21.276
Clashscore	3.19	1.79	1.64	0.6
MolProbity score	1.72	0.94	0.91	0.7
Ramachandran Plot				
Favored (%)	94	100	100	100
Allowed (%)	6	0	0	0
Outliers (%)	0	0	0	0

**Table 3:** Amino acid content (K, R, D, E, G, and P) and properties for the novel and previously characterized rubredoxins. Unusually small surface charges for the psychrophiles are marked as **bold**.

Protein	#aa	#atoms	Hydrophobic	Acidic	Basic	Neutral	#K/R	#D+E	Surface(−)	#G	#P
*Cpsy*	52	817	38.46	21.15	9.62	30.77	5/0	11	**−4.99**	6	4
*Pg*	53	806	47.17	13.21	5.66	33.96	1/1	7	**−5.04**	5	4
*Po*	55	848	32.73	25.45	9.09	32.73	3/1	14	−8.99	5	3
*Mt2b*	60	905	40.00	23.33	8.33	28.33	2/3	14	−8.99	5	3
*Pa*	55	857	41.82	23.64	9.09	25.45	4/1	13	−6.99	7	3
*Cpa*	54	808	31.48	24.07	7.41	37.04	4/1	13	−7.99	5	7
*Tm*	53	808	35.85	24.53	9.43	30.19	4/1	13	−7.99	5	7
*Pab*	53	816	35.85	24.53	11.32	28.30	4/2	13	−6.99	5	5
*Py*	53	812	39.62	24.53	9.43	26.42	4/1	13	−7.99	5	5
*Pf*	54	817	38.89	24.07	9.26	27.78	5/0	13	−7.99	5	5

## Data Availability

The original data presented in the study are openly available in the Protein Data Bank (PDB) database at https://www.rcsb.org/ under the following PDB IDs: (a) Zn *Cpsy* Rd, 9ZDO; (b) Fe *Pg* Rd, 9ZDP; (c) Fe *Py* Rd, 9ZDH; and (d) Zn *Tm* Rd, 9ZDI.

## Supplementary Materials

The following supporting information can be downloaded at: https://www.mdpi.com/article/10.3390/biom16050623/s1, Table S1: Pairwise sequence identity; Table S2: Metal–ligand distances; Table S3: Observed water molecules; Table S4: RMSD values for main chain atoms of different subunits; Table S5: Automated secondary structure assignment by STRIDE; Table S6: Protein Volume Server results; Figure S1: Rooted phylogenetic tree of Table 1 rubredoxin sequences; Figure S2: B-factor distribution; Figure S3: Normalized B-factor distribution for main chain atoms; Figure S4: Normalized B-factor distribution for all atoms; Figure S5: ProPHet rigidity web server results; Figure S6: Total interaction energy (IE) versus information content (IC) scatter plots.

## Abbreviations

The following abbreviations are used in this manuscript:

*Cpsy*	*Clostridium psychrophilum*
*Pg*	*Polaromonas glacialis*
*Tm*	*Thermotoga maritima*
*Py*	*Pyrococcus yayanosii*
*Pf*	*Pyrococcus furiosus*
*Cpa*	*Clostridium pasteurianum*
*Po*	*Pseudomonas oleovorans*
*Mt*	*Mycobacterium tuberculosis*
*Pa*	*Pseudomonas aeruginosa*
