# Supplementary material for: Crystallography of Extremophile Proteins—Structural Comparisons of Psychrophilic and Hyperthermophilic Rubredoxins
Source: Biomolecules. 2026 Apr 22;16(5):623. doi: 10.3390/biom16050623 (PMC13204472; doi:10.3390/biom16050623)
Supplement: Supplementary file 1 [file biomolecules-16-00623-s001.zip › biomolecules-4228628-supplementary.pdf]

Supplementary Materials

	Pf	***Py***	***Tm***	***Cpsy***	***Cpa***	***Pg***
Pf	**100**	77	68	60	57	49
Py	77	**100**	70	62	70	47
Tm	68	70	**100**	65	70	51
Cpsy	60	62	65	**100**	60	44
Cpa	57	70	70	60	**100**	40
Pg	49	47	51	44	40	**100**

Table S1. Pairwise sequence identity statistics for the novel four Rds and *Pf* and *Cpa* control sequences.

	Zn *Cpsy* 9ZDO	Fe *Pg* 9ZDPa	Fe *Pg* 9ZDPb	Fe *Pg* 9ZDPc	Fe *Pg* 9ZDPd	Fe *Pg* 9ZDPe	Fe *Pg* 9ZDPf	Fe *Pg* 9ZDPg	Zn *Tm* 9ZDIa	Zn *Tm* 9ZDIb	Fe Py 9ZDHa	Fe Py 9ZDHb	Fe Py 9ZDHc
Cys9(8)	2.33	**2.36**	**2.4**	**2.31**	**2.4**	2.25	2.28	2.22	2.31	2.31	2.28	**2.32**	2.27
Cys42(41)	2.3	2.25	2.24	2.27	2.23	2.34	2.26	2.29	2.31	2.32	2.28	2.27	**2.31**
Cys6(5)	2.34	2.4	2.34	2.32	2.33	2.34	2.34	2.31	2.35	2.35	2.31	2.31	**2.29**
Cys39(38)	2.36	**2.25**	**2.26**	**2.28**	2.34	2.34	2.31	2.33	2.33	2.36	2.27	**2.28**	2.3
RMSD	0.118	0.149	0.167	0.142	0.136	0.136	0.106	0.136	0.149	0.154	0.152	0.11	0.123

Table S2. Metal-ligand distances for various Rd structures and RMSD deviation from tetrahedral symmetry. Sequence numbering (-1) for Fe *Pg* Rd is given in the brackets. Bonds to Cys6 and Cys39 shielded by aromatic residues should be longer. Unusually shorter or longer distances are shown in **bold**.

**Organism**	**#aa**	**#waters**	**aa/water**	**water/aa**	**PDB_ID**	**Resolution (Å)**
Cpa	53	58	0.91	1.09	1FHH	1.50
Cpsy	52	71	0.73	1.36	9ZDO	0.84
Pg	371	227	0.61	1.63	9ZDP	1.83
Py	159	209	0.75	1.34	9ZDH	1.36
Tm	106	114	0.93	1.07	9ZDI	1.02
Pf	53	240	0.22	4.53	1BRF	0.95
Pf	54	183	0.30	3.39	5NW3	0.59
Pab	212	156	1.36	0.74	1YK5	1.79

Table S3. Observed water molecules for various Rd structures.

Pg Rd	SubunitA	SubunitB	SubunitC	SubunitD	SubunitE	SubunitF	SubunitG
SubunitA	0	0.22	0.22	0.87	0.69	0.80	0.68
SubunitB		0	0.16	0.92	0.78	0.88	0.72
SubunitC			0	0.94	0.80	0.89	0.74
SubunitD				0	0.53	0.56	0.34
SubunitE					0	0.38	0.46
SubunitF						0	0.51
SubunitG							0
Py Rd	SubunitA	SubunitB	SubunitC				
SubunitA	0	0.77	0.48				
SubunitB		0	0.41				
SubunitC			0				
Tm Rd	SubunitA	SubunitB					
SubunitA	0	0.59					
SubunitB		0					

Table S4. RMSD values for main chain atoms of different subunits of the novel Rd structures.

```
SEQ  1     MTWMCLICGWIYDEALGSPEHGIAAGTPWSQVPMNWTCPECGARKEDFEMVQM   53_Pg_psychrophile
NEW

STR         EEEETTTT EEEGGG BGGG BTTTT GGG TTTT TTTTT  GGGEEEEB    Pg_A

STR         EEEETTTT EEEGGGBBGGG BTTTT GGG TTTT TTTTT  GGGEEEEE    Pg_B

STR         EEEETTTT EEEGGG BGGG BTTTT GGG TTTTBTTTTT BGGGEEEEE    Pg_C

STR         EEEETTTT EEEGGG BGGG BTTTT GGG TTTTBTTTTT BGGGEEE      Pg_D

STR         EEEETTTT EEEGGG BGGG BTTTT GGG TTTTBTTTTT BGGGEEEE     Pg_E

STR         EEEETTTT EEETTTTBGGG BTTTT GGG TTTTBTTTTT BGGGEEEE     Pg_F

STR         EEEETTTT EEETTTTBGGG BTTTT GGG TTTTBTTTTT BGGGEEE      Pg_G

SEQ  1    MNKYVCLVCGYEYDPEIGDLEGGIKPGTKFEDLPEDWLCPLCGVTKFDFEKI     52_Cpsy_psychrophile
NEW

STR          EEETTTT EETTTTTBGGG BTTTT GGG TTTTBTTTTT BGGGEEE      Cpsy_A

SEQ  1    MKKYTCTVCGYIYNPEDGDPDNGVNPGTDFKDIPDDWVCPLCGVGKDQFEEVE    53_Cpa_1FHH_mesophile
CONTROL

STR          EEETTTT EETTTTTBGGG BTTTT GGG TTTTBTTTTT BGGGEEE      Cpa_1FHH

SEQ  1    MKKYRCKLCGYIYDPEQGDPDSGIEPGTPFEDLPDDWVCPLCGASKEDFEPV     52_Tm_hyperthermophile
NEW

STR          EEETTTT EE GGG BGGG BTTTT GGG TTTTBTTTTT BGGGEEE      Tm_A

STR          EEETTTT EE GGG BGGG BTTTT GGG TTTTBTTTTT BGGGEEE      Tm_B
```

```
SEQ  1     AKWRCTVCGYIYDEEEGDPDNGVLPGTKFEELPDDWVCPLCGAPKDMFEKVD
53_Py_hyperthermophile_piesophile     NEW

STR         EEEETTTT EEEGGG BGGG BTTTT GGG TTTT TTTTT  GGGEEEE     Py_A

STR         EEEETTTT EEEGGG BGGG BTTTT GGG TTTT TTTTT  GGGEEEE     Py_B

STR         EEEETTTT EEEGGG BGGG BTTTT GGG TTTT TTTTT  GGGEEE      Py_C

SEQ  1    MAKWRCKICGYIYDEDEGDPDNGISPGTKFEDLPDDWVCPLCGAPKSEFERIE
53_Pa_1YK5_hyperthermophile_piesophile

STR         EEEETTTT EEETTTTBGGG BTTTT GGG TTTT TTTTT  GGGEEE      Pa_A_1YK5

STR         EEEETTTT EEETTTTBGGG BTTTT GGG TTTT TTTTT  GGGEEE      Pa_B_1YK5

STR         EEEETTTT EEETTTTBGGG BTTTT GGG TTTT TTTTT  GGGEEE      Pa_C_1YK5

STR         EEEETTTT EEETTTTBGGG BTTTT GGG TTTT TTTTT  GGGEEE      Pa_D_1YK5

SEQ  1    MAKWVCKICGYIYDEDAGDPDNGISPGTKFEELPDDWVCPICGAPKSEFEKLED
54_Pf_5NW3_hyperthermophile

STR         EEEETTTT EEEGGG BGGG BTTTT GGG TTTT TTTTT  GGGEEETT    Pf_A_5NW3
```

Table S5. Automated secondary structure assignment by *STRIDE* for all rubredoxin subunit of the 4 novel and already reported *Cpa*, *Pf*, and *Pab* rubredoxins. Slight variations are observed. **Stride Legend**: **H**=Alpha helix; **G**= 3-10 helix; **I**= PI-helix; **E**= Extended conformation (beta strand); **B** or **b**= Isolated bridge; **T**= Turn; **C**= Coil (none of the above).

Protein	Density	VdW_Volume	TotalVolume	VoidVolume	#aa	%solvent	Note
*Cpsy*_sg19	**0.782**	5272.99	6746.68	1473.69	52	**30.1**	
*Pg*_sg96a	**0.780**	5268.14	6755.29	1487.15	53	62.2	
*Pg*_sg96b	**0.781**	5262.68	6735.86	1473.18	53	62.2	
*Pg*_sg96c	**0.778**	5270.30	6772.20	1501.90	53	62.2	
*Pg*_sg96d	**0.788**	5267.88	6682.39	1414.45	53	62.2	
*Pg*_sg96e	**0.780**	5261.87	6743.06	1481.19	53	62.2	
*Pg*_sg96f	**0.787**	5266.25	6687.55	1421.30	53	62.2	
*Pg*_sg96g	**0.779**	5276.03	6775.99	1499.96	53	62.2	
*Cpa*_1FHH	0.788	5162.19	6549.46	1387.26	54	41.5	
*Tm*_sg4a	**0.785**	5131.70	6533.06	1401.35	52	**31.8**	
*Tm*_sg4b	**0.778**	5141.13	6607.70	1466.56	52	**31.8**	
*Py*_sg19a	**0.784**	5165.37	6592.31	1426.94	52	**35.4**	
*Py*_sg19b	**0.784**	5165.30	6585.06	1419.76	52	**35.4**	
*Py*_sg19c	**0.785**	5162.96	6580.52	1417.55	52	**35.4**	
*Pf*_1BRF	0.786	5186.57	6602.80	1416.23	53	44.1	(0.95 Å)
*Pf*_5NW3	0.792	5292.55	6681.78	1389.23	54	40.7	(0.59 Å)
*Pf*_Fe2_5OME	0.793	5295.58	6678.88	1383.30	54	42.0	(0.75 Å)
*Pab*_1YK5_A	0.774	5297.16	6843.91	1546.76	52	55.0	(286K, RT)
*Pab*_1YK5_B	0.776	5299.08	6828.21	1529.13	52	55.0	(286K, RT)
*Pab*_1YK5_C	0.774	5185.69	6701.40	1515.72	52	55.0	(286K, RT)
*Pab*_1YK5_D	0.774	5302.49	6850.66	1548.17	52	55.0	(286K, RT)
*Pab*_1YK4	0.787	5067.62	6437.88	1370.25	53	36.5	(100K, mutant)

Table S6. Protein Volume Server results for Density, VdW, Total, and Void Volumes for the 4 novel and already reported *Cpa*, *Pf*, and *Pab* rubredoxins [38]. No systematic differences were observed based on temperature adaptation criteria.

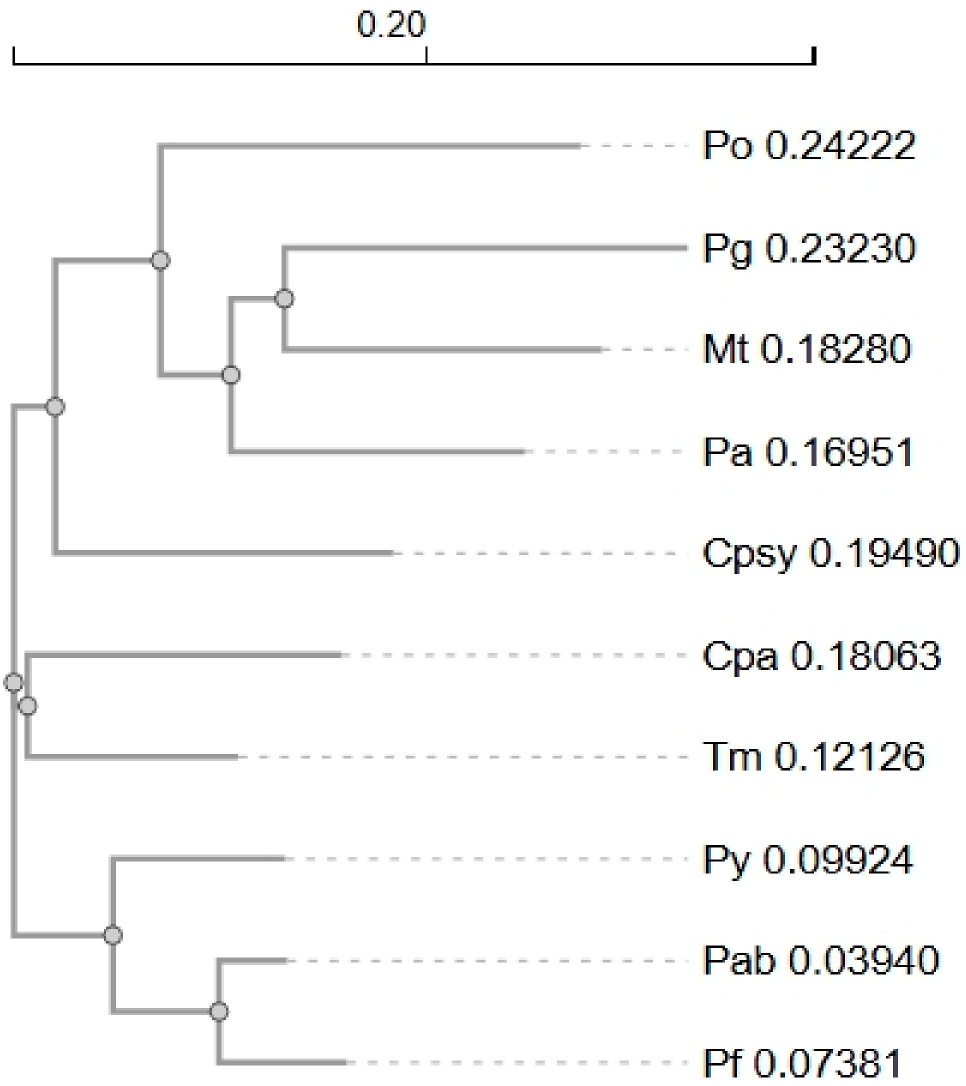

Figure S1. Rooted phylogenetic tree of the **Table 1** rubredoxin sequences. There is a clear separation of the aerobic organisms on the top of the tree: *Po*, *Pg*, *Mt*, and *Pa*, and the anaerobic organisms at the bottom: *Pf*, *Pab*, *Py*, *Cpa*, *Tm*, *Cpsy*. One of the psychrophilic rubredoxins (*Cpsy*) is in the anaerobic branch of the tree.

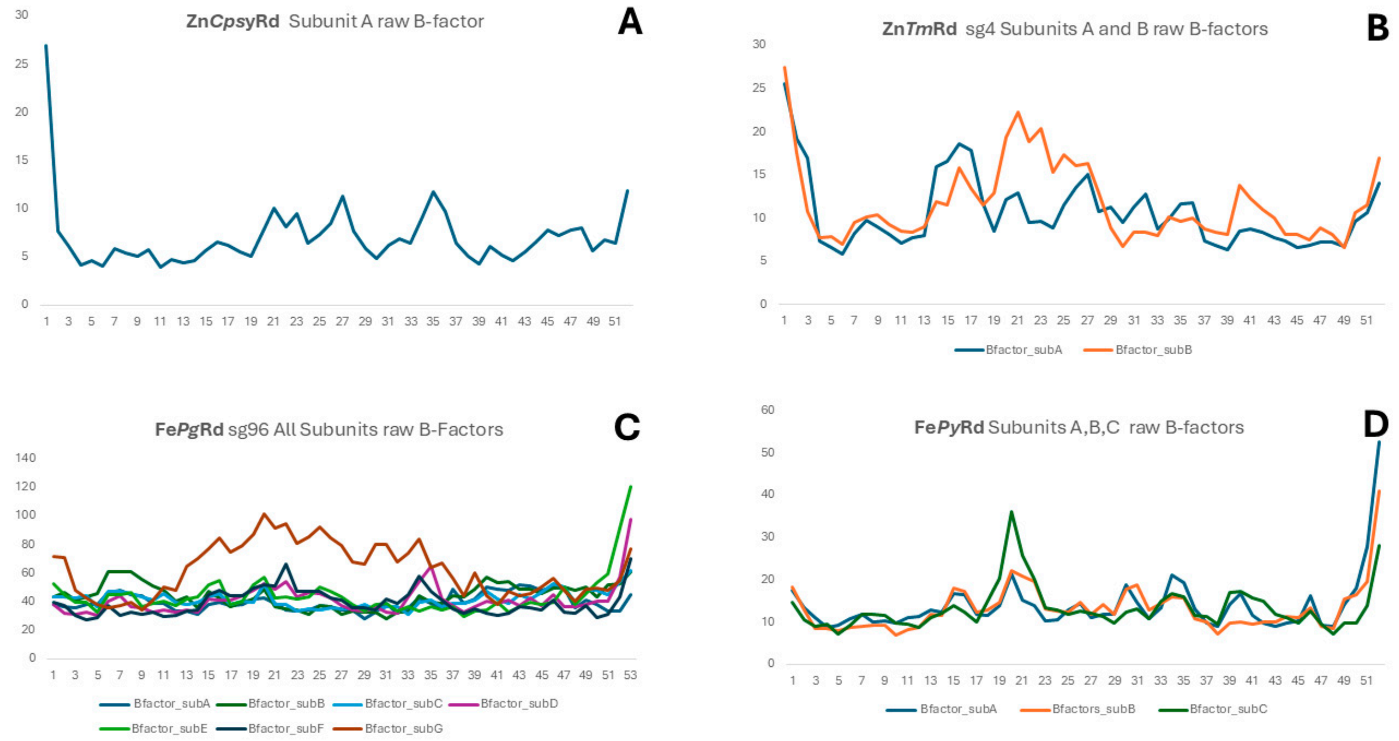

Figure S2. B-factor distribution for the new 4 experimental structures by the BANDIT web server. (**A**) Zn *Cpsy* Rd, (**B**) Zn *Tm* Rd, (**C**) Fe *Pg* Rd, (**D**) Fe *Py* Rd. Note the wide range for the B-factors distribution for the different Fe *Pg* Rd subunits.

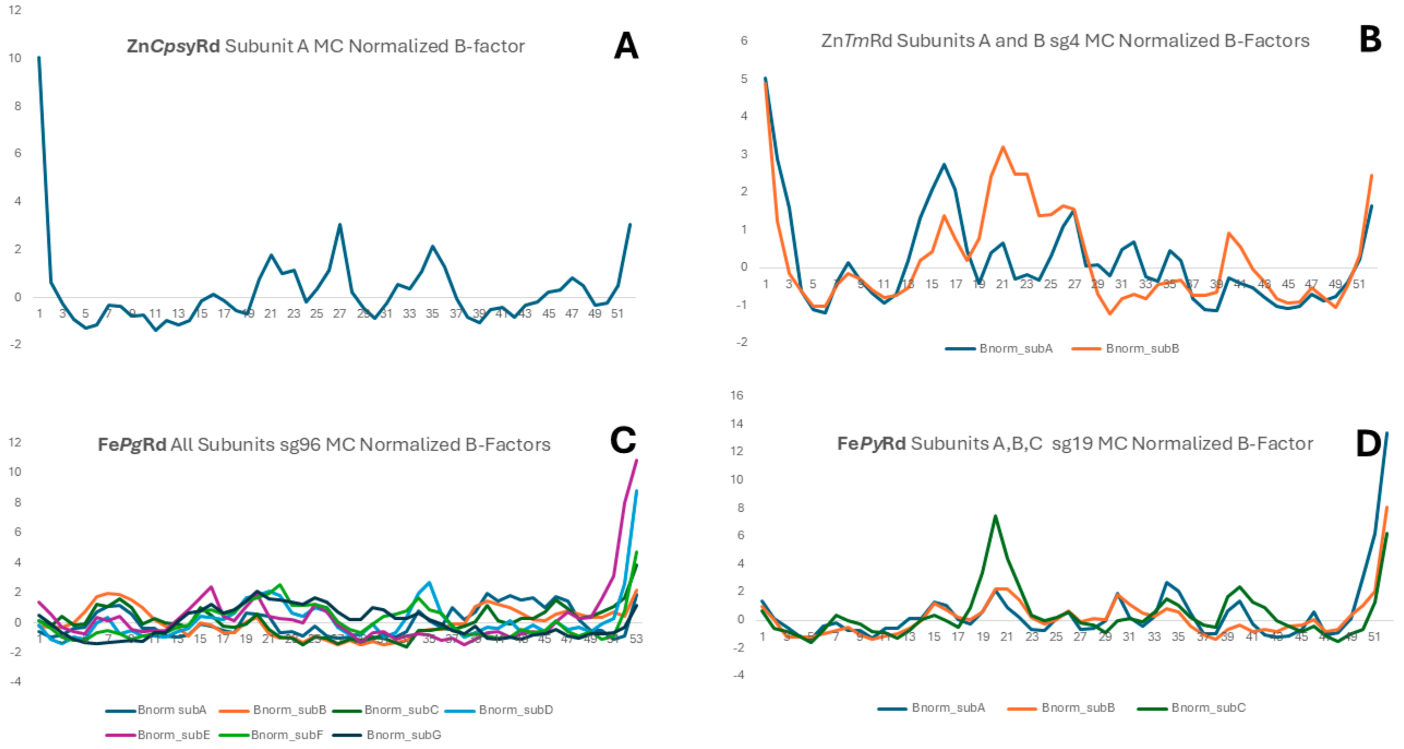

Figure S3. Normalized B-factor distribution for main chain atoms for the new 4 experimental structures by the BANDIT web server (**A**) Zn *Cpsy* Rd, (**B**) Zn *Tm* Rd, (**C**) Fe *Pg* Rd, (**D**) Fe *Py* Rd.

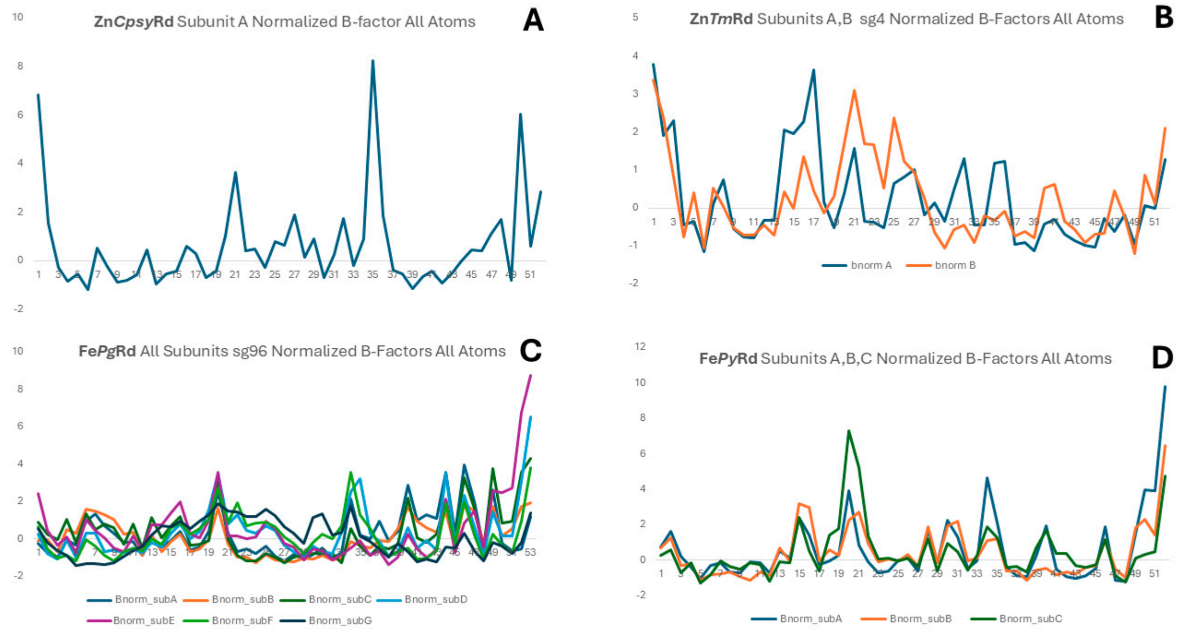

Figure S4. Normalized B-factor distribution for all atoms for the new 4 experimental structures by the BANDIT web server. (**A**) Zn *Cpsy* Rd, (**B**) Zn *Tm* Rd, (**C**) Fe *Pg* Rd, (**D**) Fe *Py* Rd.

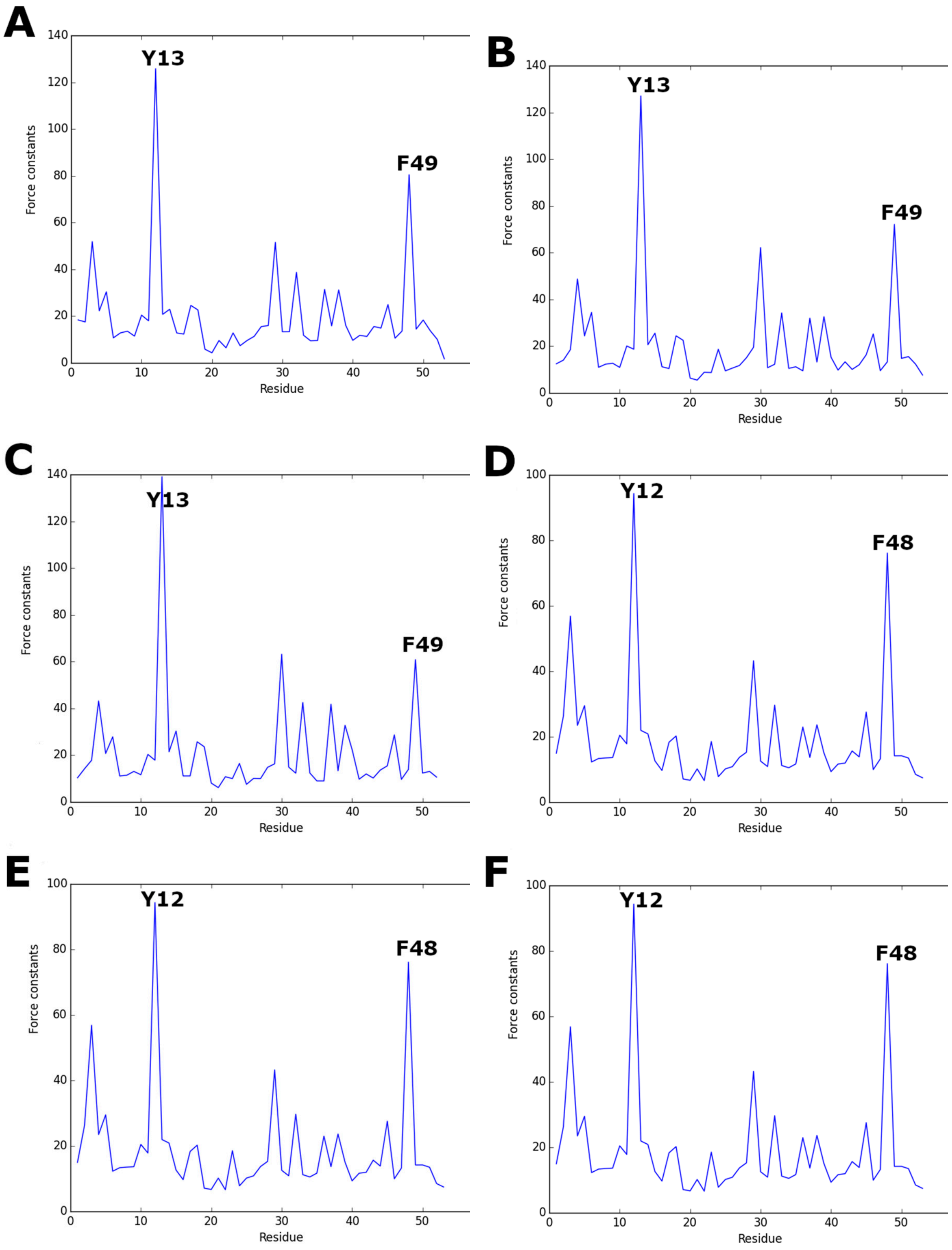
A
B
C
D
E
F
Y13
F49
Y12
F48
Force constants
Residue

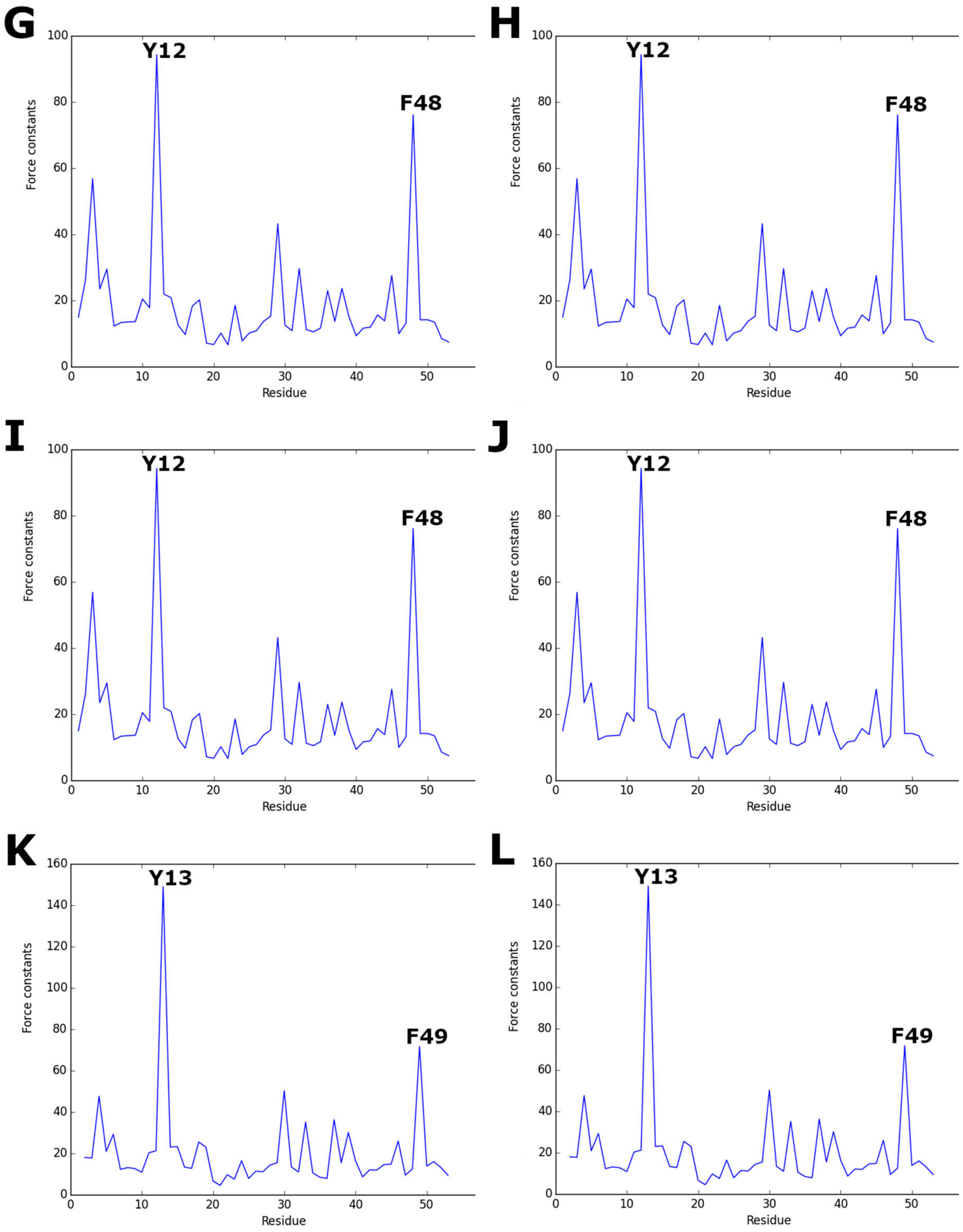
G
Y12
F48
Force constants
Residue
H
Y12
F48
Force constants
Residue
I
Y12
F48
Force constants
Residue
J
Y12
F48
Force constants
Residue
K
Y13
F49
Force constants
Residue
L
Y13
F49
Force constants
Residue

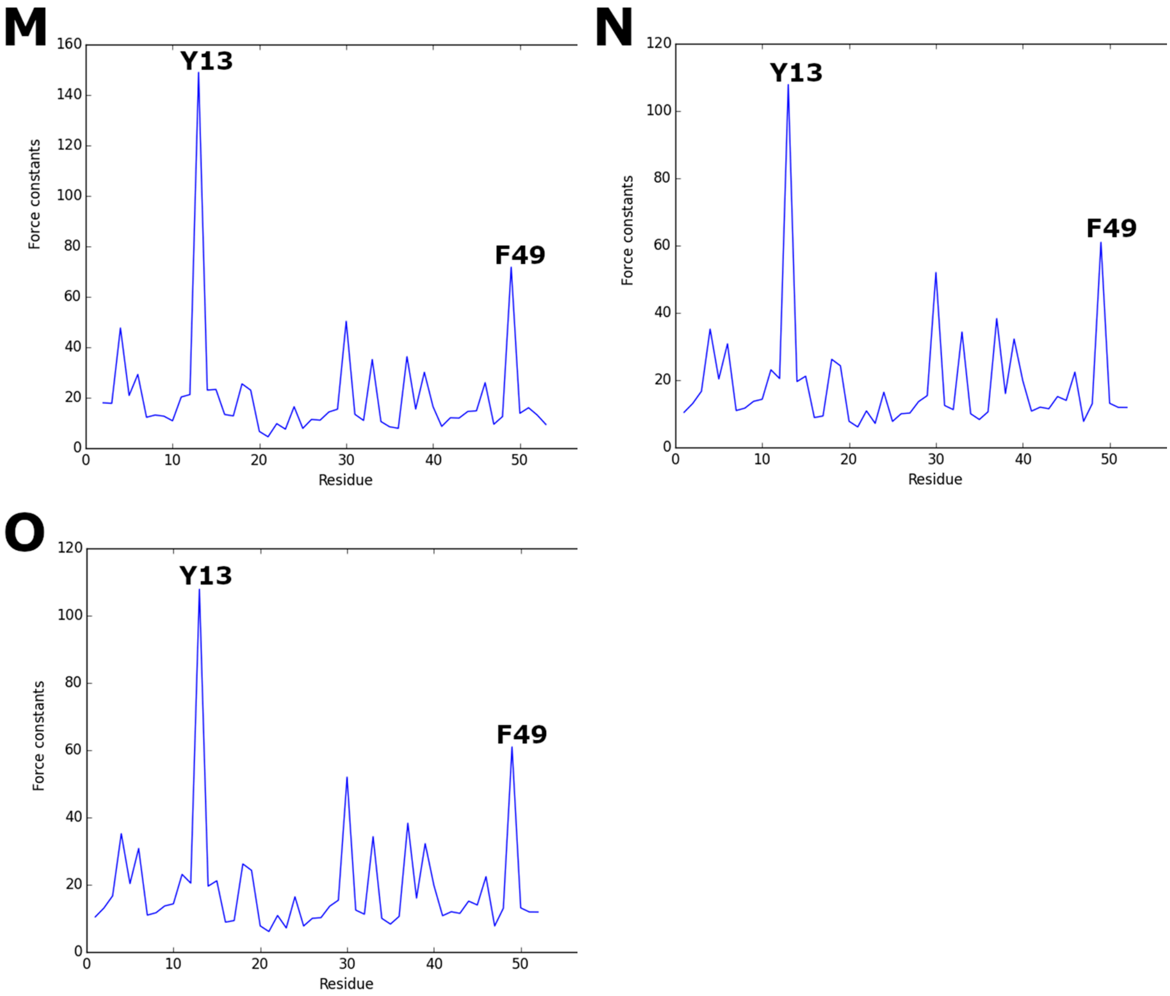

Figure S5. ProPHet rigidity web server confirming the strongest mechanical interactions for holding the rubredoxin fold in all novel rubredoxin subunits as well as the Fe *Pf* Rd (1BRF) and Fe *Cpa* Rd (1FHH) control structures involve buried aromatic residues (Y13, F49). Note the overall lower absolute peaks (Y12, F48) for all the Fe *Pg* Rd subunits (**D** to **J**) as well as the increased rigidity at W3 (W4/Y4 in the other rubredoxins). *Cpsy* rigidity peaks are in the range with the thermophilic residues (Y13> 120), probably related to high resolution and low solvent content. **A**. Fe *Pf* Rd sg19. **B**. Fe *Cpa* Rd(1FHH). **C**. Zn *Cpsy* Rd. **D**. Fe *Pg* Rd_subunit A, **E**. Fe *Pg* Rd_subunit B, **F.** Fe *Pg* Rd_subunit C, **G**. Fe *Pg* Rd_subunit D, **H**. Fe *Pg* Rd_subunit E, **I**. Fe *Pg* Rd_subunit F, **J**. Fe *Pg* Rd_subunit G, **K**. Fe *Py* Rd_subunit A, **L**. Fe *Py* Rd_subunit B, **M**. Fe *Py* Rd_subunit C, **N**. Zn *Tm* Rd_subunit A, **O**. Zn *Tm* Rd_subunit B.

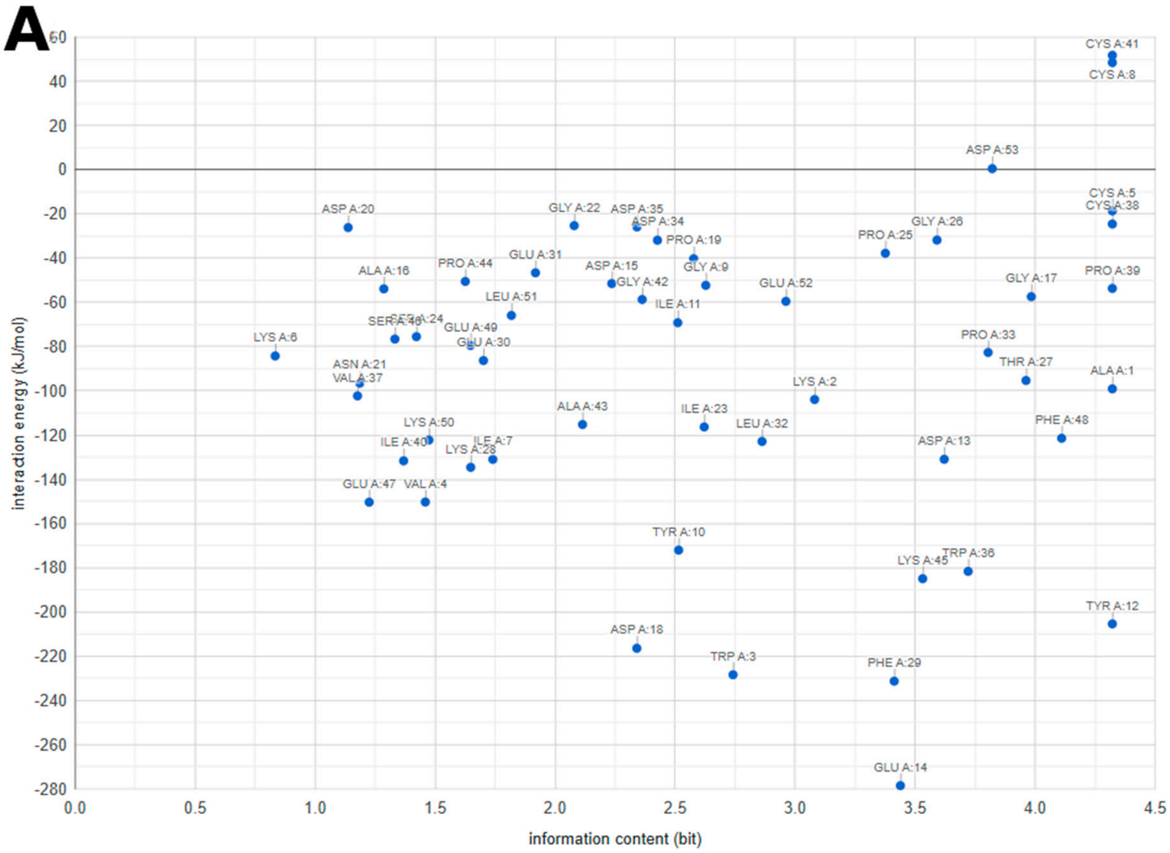
A
interaction energy (kJ/mol)
information content (bit)
CYS A:41
CYS A:8
ASP A:53
CYS A:5
CYS A:38
ASP A:20
GLY A:22
ASP A:35
ASP A:34
PRO A:19
GLY A:26
PRO A:25
GLU A:31
PRO A:44
ALA A:16
ASP A:15
GLY A:9
GLY A:42
GLU A:52
GLY A:17
PRO A:39
LEU A:51
ILE A:11
GLU A:49
GLU A:30
LYS A:6
PRO A:33
THR A:27
ASN A:21
VAL A:37
LYS A:2
ALA A:1
ALA A:43
ILE A:23
LEU A:32
LYS A:50
PHE A:48
ILE A:40
ILE A:7
LYS A:28
ASP A:13
GLU A:47
VAL A:4
TYR A:10
LYS A:45
TRP A:36
TYR A:12
ASP A:18
TRP A:3
PHE A:29
GLU A:14

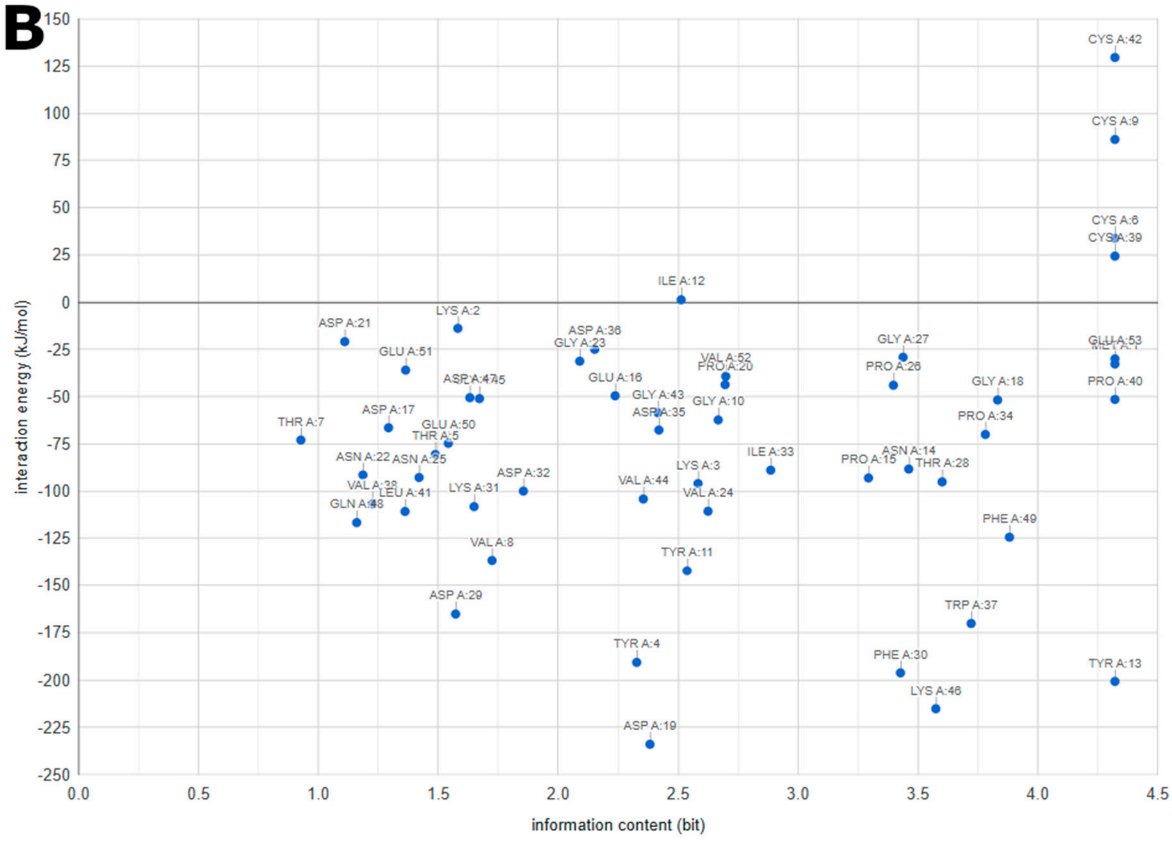
B
interaction energy (kJ/mol)
information content (bit)
CYS A:42
CYS A:9
CYS A:6
CYS A:39
ILE A:12
LYS A:2
ASP A:21
ASP A:36
GLY A:23
GLY A:27
GLU A:51
VAL A:52
PRO A:20
PRO A:26
GLU A:16
GLY A:18
PRO A:40
GLY A:43
GLY A:10
ASP A:35
ASP A:17
GLU A:50
THR A:7
THR A:5
PRO A:34
ASN A:22
ASN A:25
ILE A:33
ASN A:14
PRO A:15
THR A:28
ASP A:32
LYS A:3
VAL A:44
VAL A:24
LEU A:41
GLN A:48
LYS A:31
PHE A:49
VAL A:8
TYR A:11
ASP A:29
TRP A:37
TYR A:4
PHE A:30
TYR A:13
LYS A:46
ASP A:19

C

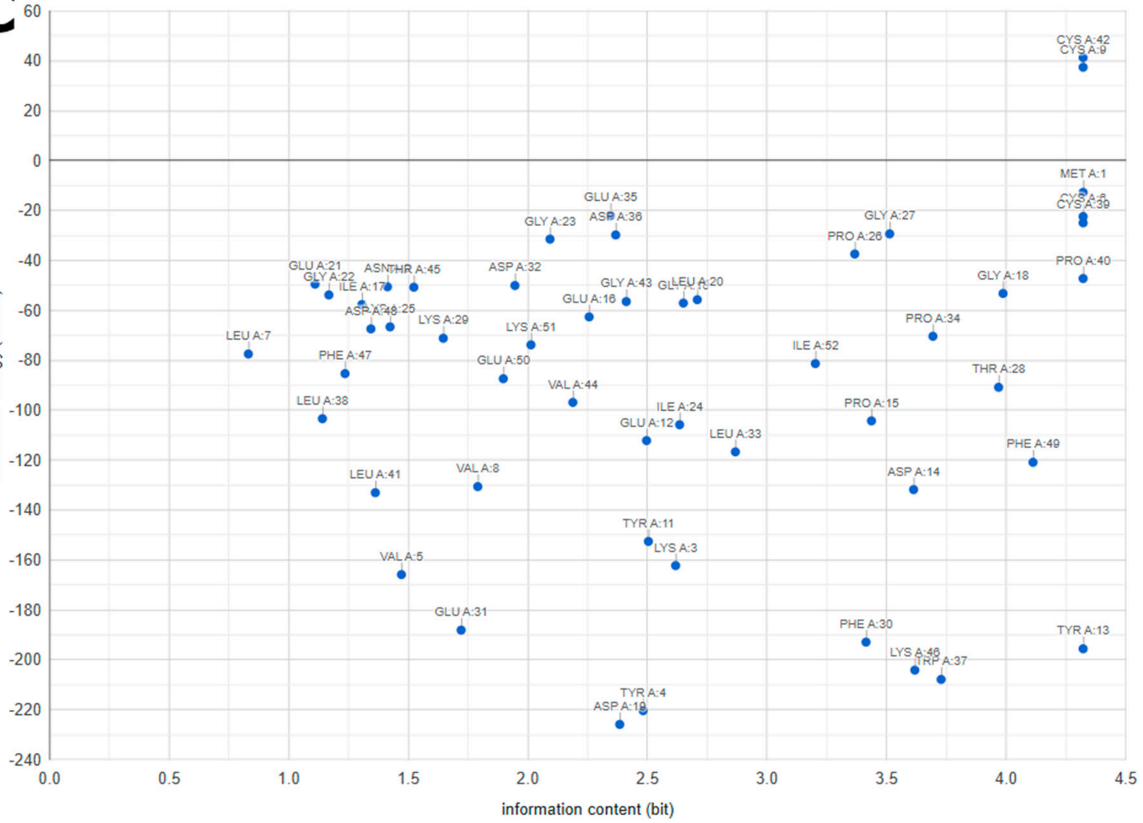
interaction energy (kJ/mol)
information content (bit)

D

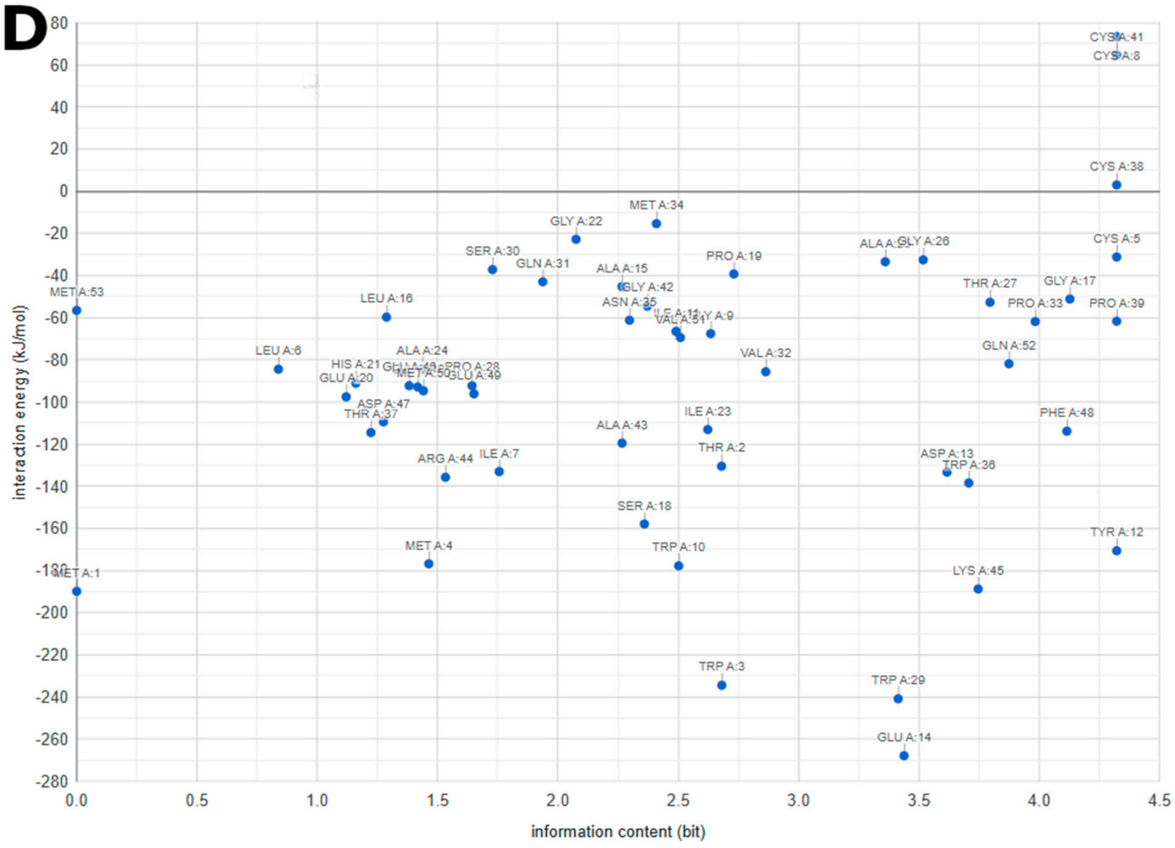
interaction energy (kJ/mol)
information content (bit)

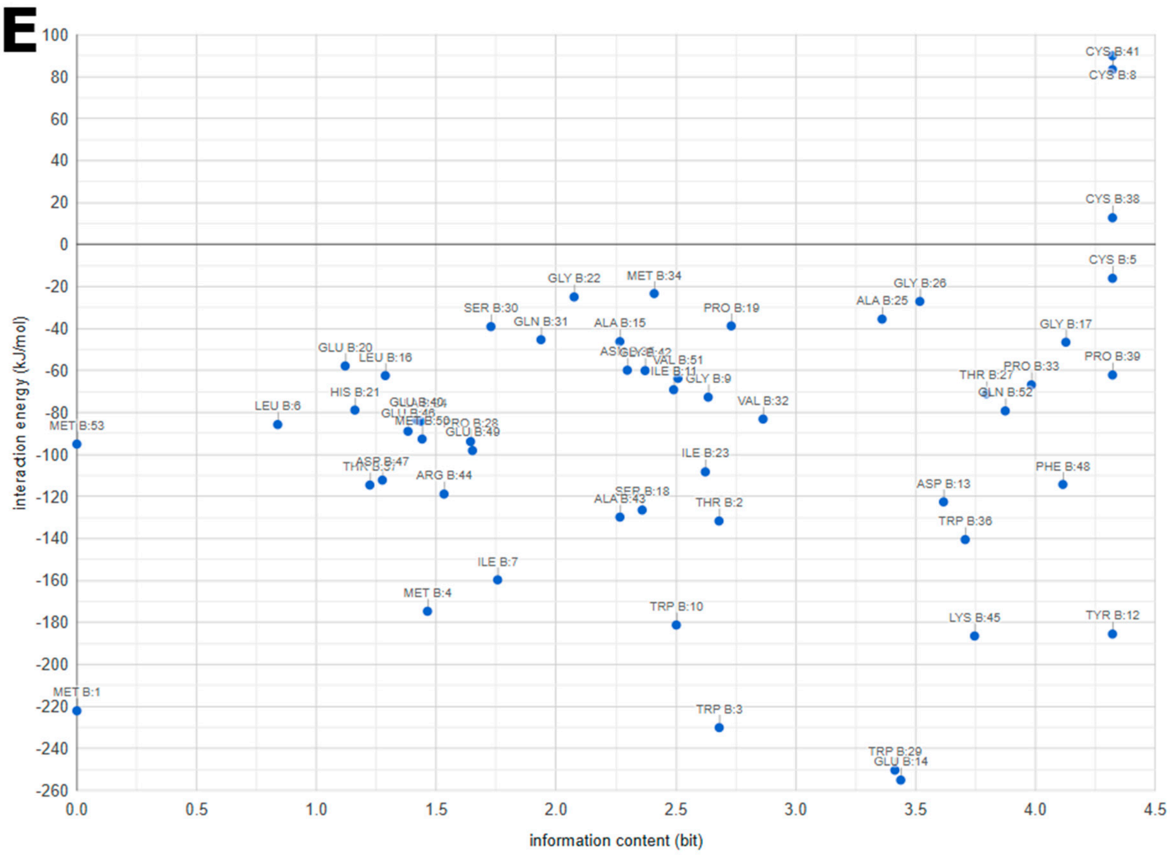
E
interaction energy (kJ/mol)
information content (bit)
100
80
60
40
20
0
-20
-40
-60
-80
-100
-120
-140
-160
-180
-200
-220
-240
-260
0.0
0.5
1.0
1.5
2.0
2.5
3.0
3.5
4.0
4.5
CYS B:41
CYS B:8
CYS B:38
CYS B:5
GLY B:22
MET B:34
GLY B:26
SER B:30
GLN B:31
ALA B:15
PRO B:19
ALA B:25
GLY B:17
GLU B:20
LEU B:16
VAL B:51
ILE B:11
GLY B:9
PRO B:33
PRO B:39
THR B:27
GLN B:52
HIS B:21
LEU B:6
MET B:53
MET B:50
PRO B:28
GLU B:49
VAL B:32
ILE B:23
ASP B:47
ARG B:44
PHE B:48
SER B:18
ALA B:43
THR B:2
ASP B:13
TRP B:36
ILE B:7
MET B:4
TRP B:10
LYS B:45
TYR B:12
MET B:1
TRP B:3
TRP B:29
GLU B:14

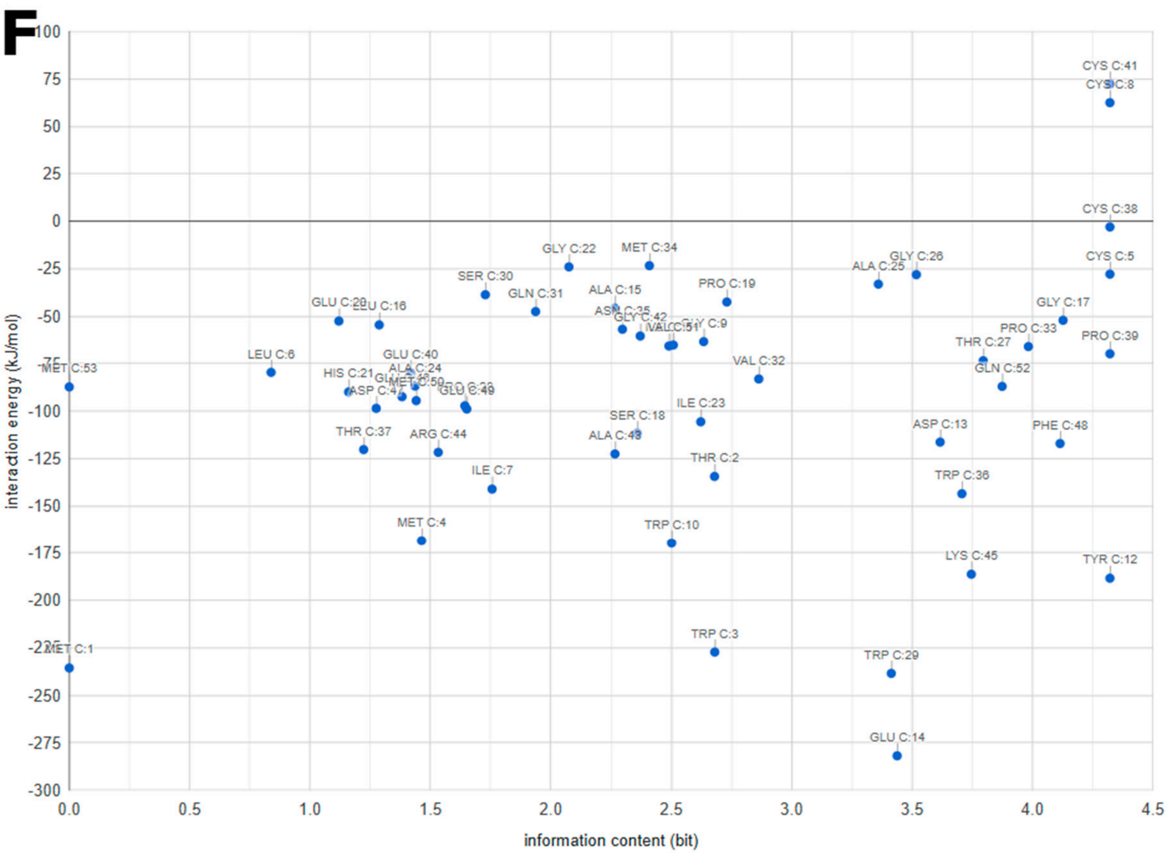
F
interaction energy (kJ/mol)
information content (bit)
100
75
50
25
0
-25
-50
-100
-125
-150
-175
-200
-250
-275
-300
0.0
0.5
1.0
1.5
2.0
2.5
3.0
3.5
4.0
4.5
CYS C:41
CYS C:8
CYS C:38
CYS C:5
GLY C:22
MET C:34
GLY C:26
ALA C:25
SER C:30
GLN C:31
ALA C:15
PRO C:19
GLY C:17
LEU C:16
GLY C:42
GLY C:9
PRO C:33
PRO C:39
THR C:27
GLN C:52
LEU C:6
GLU C:40
ALA C:24
HIS C:21
MET C:50
GLU C:49
VAL C:32
MET C:53
SER C:18
ILE C:23
ALA C:43
THR C:37
ARG C:44
ASP C:13
PHE C:48
THR C:2
TRP C:36
ILE C:7
MET C:4
TRP C:10
LYS C:45
TYR C:12
TRP C:3
MET C:1
TRP C:29
GLU C:14

G

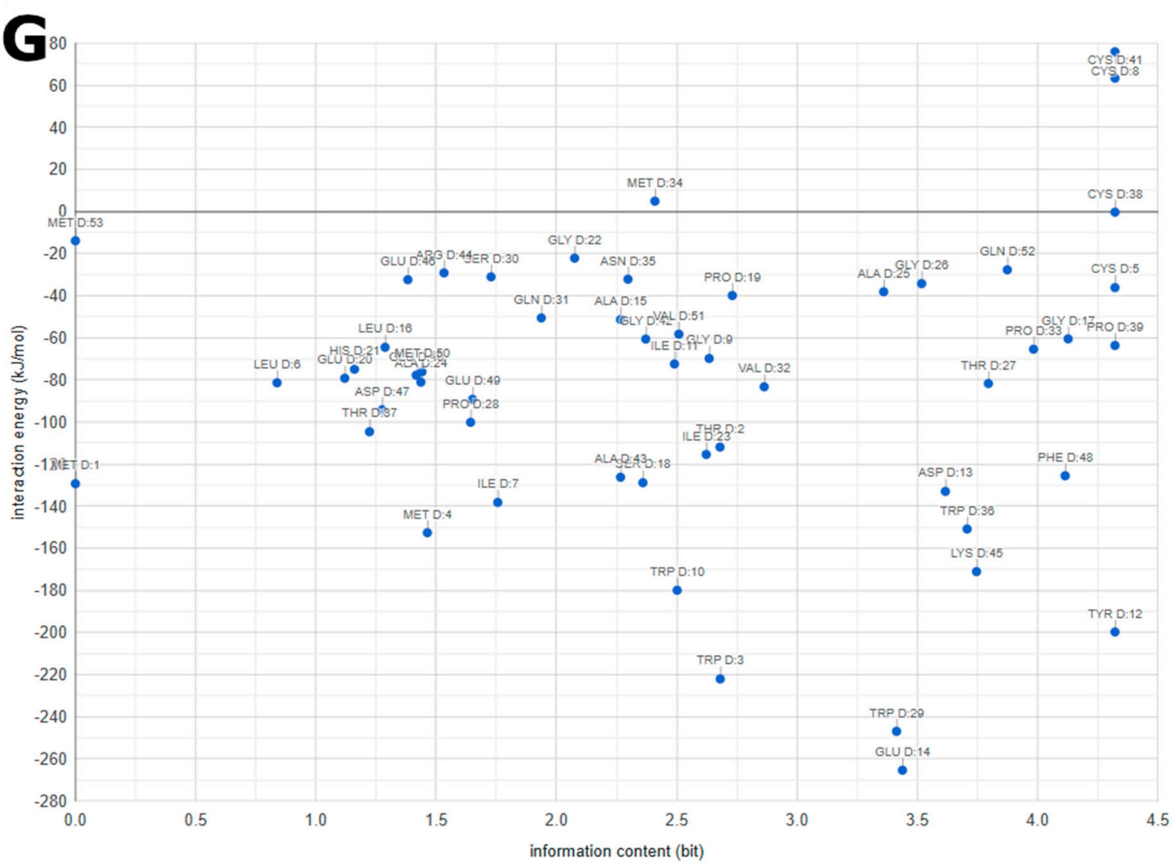

interaction energy (kJ/mol)
information content (bit)
CYS D:41
CYS D:8
MET D:34
CYS D:38
MET D:53
GLY D:22
GLN D:52
GLU D:46
ARG D:44
SER D:30
ASN D:35
GLY D:26
CYS D:5
ALA D:25
PRO D:19
GLN D:31
ALA D:15
VAL D:51
GLY D:42
GLY D:17
PRO D:33
PRO D:39
LEU D:16
ILE D:11
GLY D:9
HIS D:21
MET D:50
GLU D:20
ALA D:24
LEU D:6
THR D:27
VAL D:32
GLU D:49
ASP D:47
PRO D:28
THR D:37
THR D:2
ILE D:23
MET D:1
ALA D:43
SER D:18
PHE D:48
ASP D:13
ILE D:7
MET D:4
TRP D:36
LYS D:45
TRP D:10
TYR D:12
TRP D:3
TRP D:29
GLU D:14

H

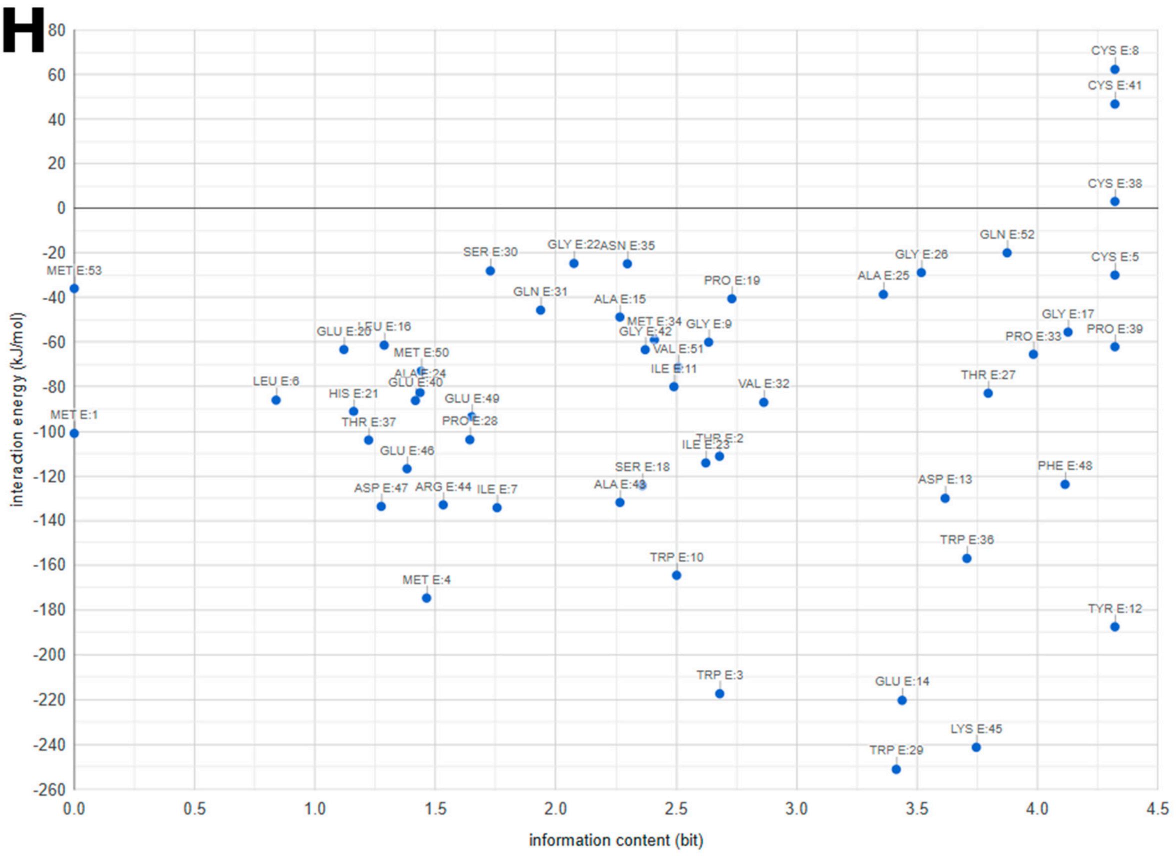

interaction energy (kJ/mol)
information content (bit)
CYS E:8
CYS E:41
CYS E:38
GLN E:52
GLY E:22
ASN E:35
SER E:30
GLY E:26
CYS E:5
MET E:53
ALA E:25
PRO E:19
GLN E:31
ALA E:15
GLY E:17
LEU E:16
MET E:34
GLY E:9
PRO E:39
GLU E:20
GLY E:42
PRO E:33
MET E:50
VAL E:51
ILE E:11
ALA E:24
THR E:27
LEU E:6
GLU E:40
VAL E:32
HIS E:21
GLU E:49
MET E:1
THR E:37
PRO E:28
THR E:2
ILE E:23
GLU E:46
SER E:18
PHE E:48
ASP E:47
ARG E:44
ILE E:7
ALA E:43
ASP E:13
TRP E:36
TRP E:10
MET E:4
TYR E:12
TRP E:3
GLU E:14
LYS E:45
TRP E:29

I

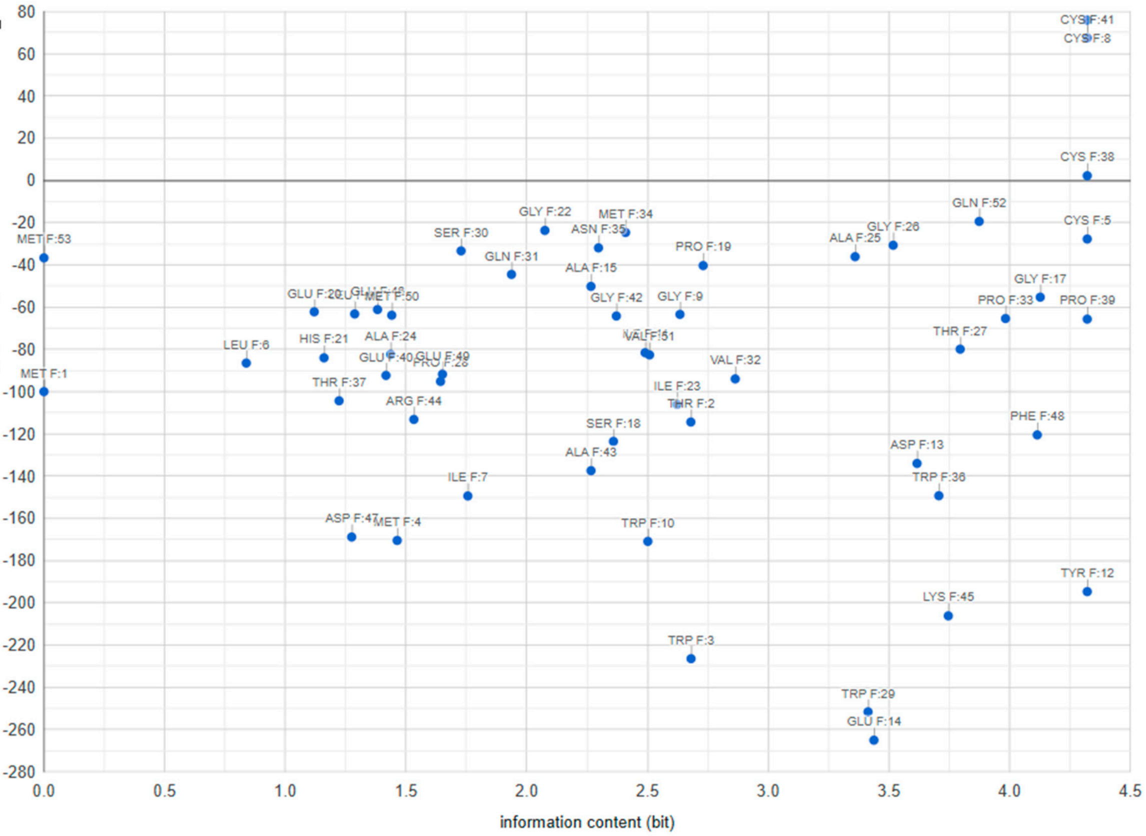
interaction energy (kJ/mol)
information content (bit)
CYS F:41
CYS F:8
CYS F:38
GLN F:52
CYS F:5
GLY F:26
ALA F:25
GLY F:22
MET F:34
ASN F:35
SER F:30
GLN F:31
PRO F:19
ALA F:15
MET F:53
GLY F:17
PRO F:33
PRO F:39
GLY F:42
GLY F:9
GLU F:20
MET F:50
THR F:27
LEU F:6
HIS F:21
ALA F:24
VAL F:51
VAL F:32
MET F:1
GLU F:40
GLU F:49
PRO F:28
THR F:37
ILE F:23
THR F:2
ARG F:44
SER F:18
PHE F:48
ALA F:43
ASP F:13
ILE F:7
TRP F:36
ASP F:47
MET F:4
TRP F:10
TYR F:12
LYS F:45
TRP F:3
TRP F:29
GLU F:14

J

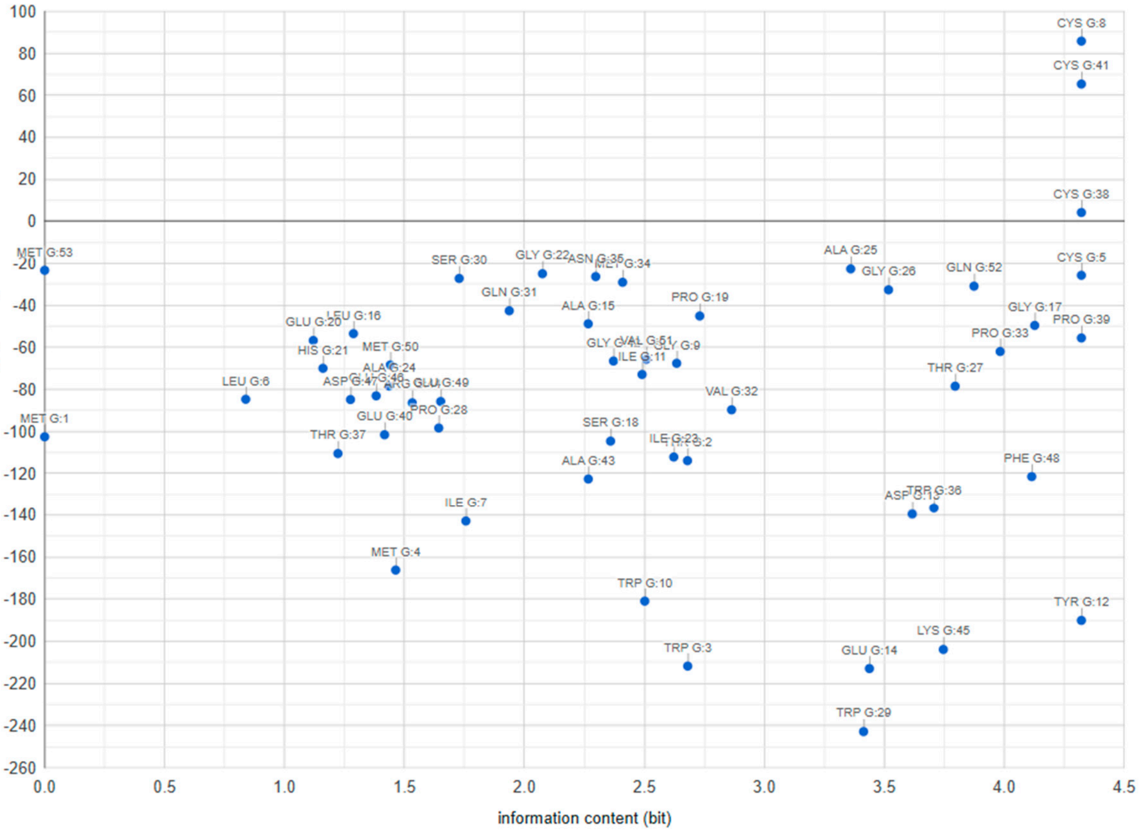
interaction energy (kJ/mol)
information content (bit)
CYS G:8
CYS G:41
CYS G:38
MET G:53
SER G:30
GLY G:22
ASN G:35
MET G:34
ALA G:25
GLY G:26
GLN G:52
CYS G:5
GLN G:31
ALA G:15
PRO G:19
GLU G:20
LEU G:16
GLY G:17
PRO G:39
MET G:50
PRO G:33
HIS G:21
VAL G:51
GLY G:9
ILE G:11
ALA G:24
THR G:27
LEU G:6
ASP G:47
GLU G:49
VAL G:32
MET G:1
GLU G:40
PRO G:28
THR G:37
SER G:18
ILE G:23
ALA G:43
PHE G:48
TRP G:36
ILE G:7
MET G:4
TRP G:10
TYR G:12
TRP G:3
LYS G:45
GLU G:14
TRP G:29

K

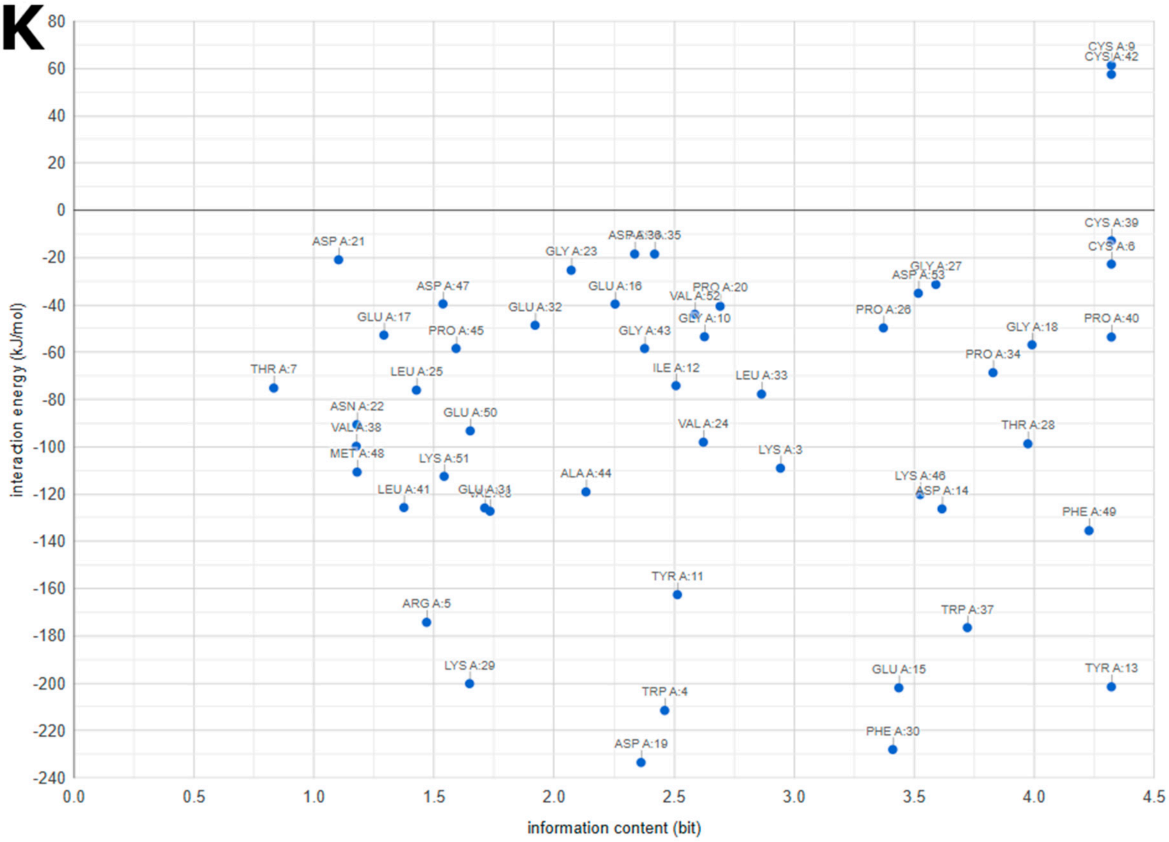
interaction energy (kJ/mol)
information content (bit)
80
60
40
20
0
-20
-40
-60
-80
-100
-120
-140
-160
-180
-200
-220
-240
0.0
0.5
1.0
1.5
2.0
2.5
3.0
3.5
4.0
4.5
CYS A:9
CYS A:42
CYS A:39
CYS A:6
ASP A:21
GLY A:23
GLY A:27
ASP A:53
ASP A:47
GLU A:16
PRO A:20
VAL A:52
GLU A:32
GLY A:10
PRO A:26
GLU A:17
PRO A:45
GLY A:43
GLY A:18
PRO A:40
PRO A:34
THR A:7
LEU A:25
ILE A:12
LEU A:33
ASN A:22
GLU A:50
VAL A:38
VAL A:24
THR A:28
MET A:48
LYS A:51
LYS A:3
LYS A:46
ALA A:44
LEU A:41
ASP A:14
PHE A:49
TYR A:11
ARG A:5
TRP A:37
LYS A:29
GLU A:15
TYR A:13
TRP A:4
PHE A:30
ASP A:19

L

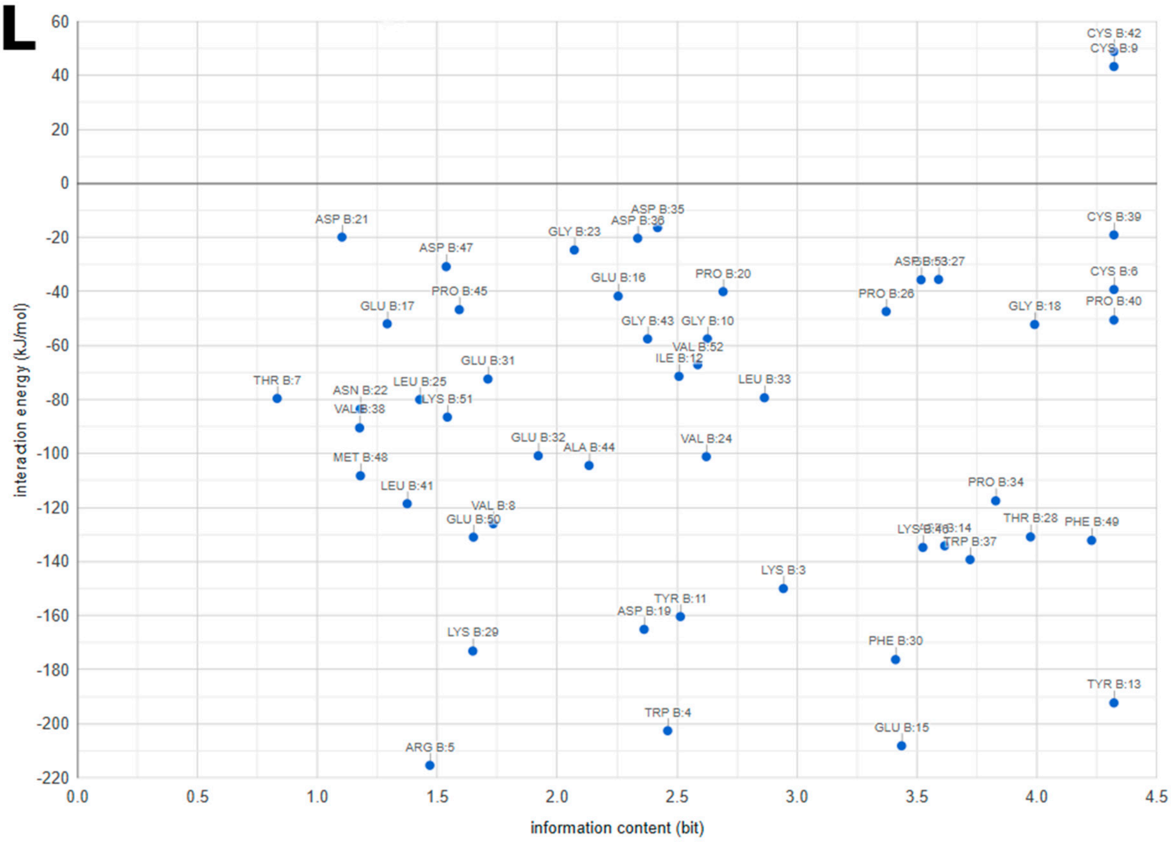
interaction energy (kJ/mol)
information content (bit)
60
40
20
0
-20
-40
-60
-80
-100
-120
-140
-160
-180
-200
-220
0.0
0.5
1.0
1.5
2.0
2.5
3.0
3.5
4.0
4.5
CYS B:42
CYS B:9
ASP B:35
ASP B:21
ASP B:36
CYS B:39
GLY B:23
ASP B:47
CYS B:6
PRO B:20
GLU B:16
PRO B:26
PRO B:40
PRO B:45
GLU B:17
GLY B:18
GLY B:43
GLY B:10
VAL B:52
GLU B:31
ILE B:12
THR B:7
LEU B:25
LEU B:33
ASN B:22
LYS B:51
VAL B:38
GLU B:32
ALA B:44
VAL B:24
MET B:48
PRO B:34
LEU B:41
VAL B:8
THR B:28
PHE B:49
GLU B:50
TRP B:37
LYS B:3
TYR B:11
ASP B:19
LYS B:29
PHE B:30
TYR B:13
TRP B:4
GLU B:15
ARG B:5

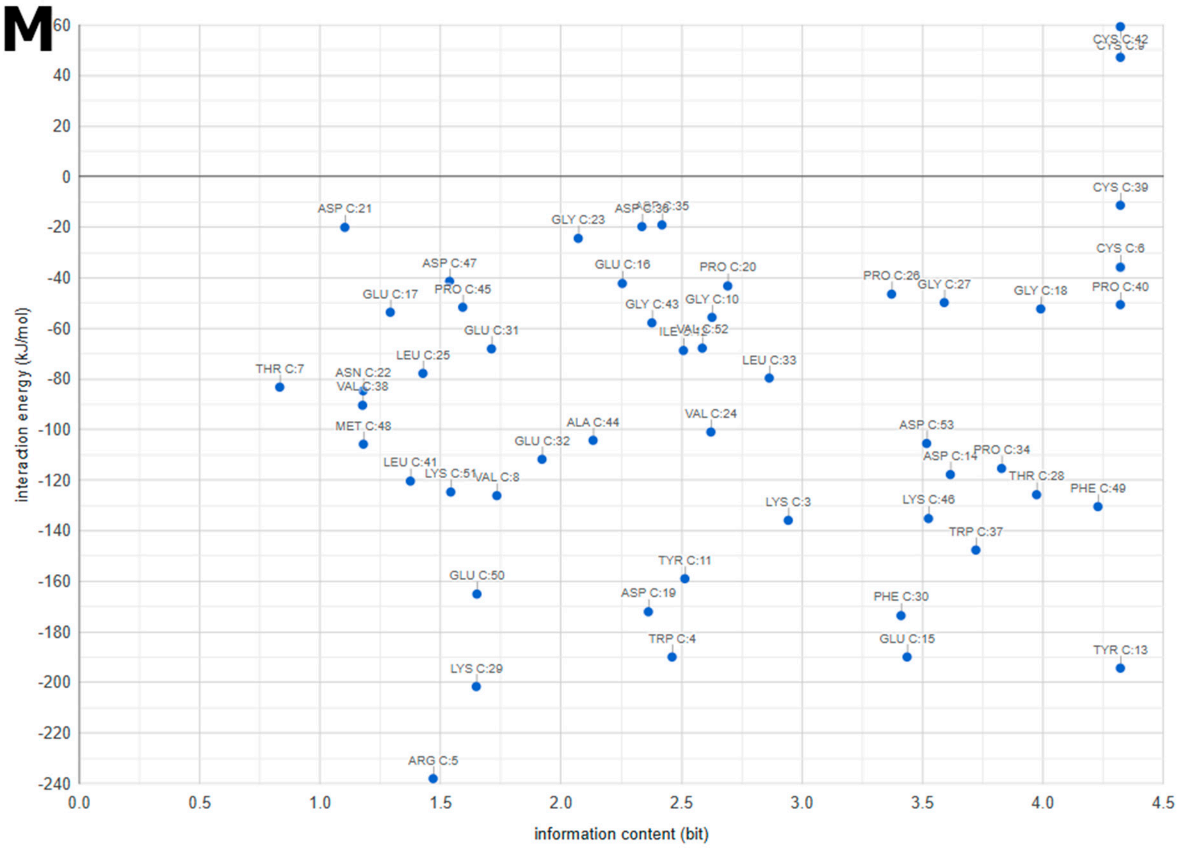
M
interaction energy (kJ/mol)
information content (bit)
60
40
20
0
-20
-40
-60
-80
-100
-120
-140
-160
-180
-200
-220
-240
0.0
0.5
1.0
1.5
2.0
2.5
3.0
3.5
4.0
4.5
CYS C:42
CYS C:39
CYS C:6
PRO C:40
ASP C:21
GLY C:23
ASP C:36
ASP C:47
GLU C:16
PRO C:20
PRO C:26
GLY C:27
GLY C:18
GLU C:17
PRO C:45
GLY C:43
GLY C:10
GLU C:31
VAL C:52
LEU C:25
LEU C:33
THR C:7
ASN C:22
VAL C:38
MET C:48
ALA C:44
VAL C:24
GLU C:32
ASP C:53
ASP C:14
PRO C:34
LEU C:41
LYS C:51
VAL C:8
THR C:28
PHE C:49
LYS C:3
LYS C:46
TRP C:37
TYR C:11
GLU C:50
ASP C:19
PHE C:30
TRP C:4
GLU C:15
TYR C:13
LYS C:29
ARG C:5

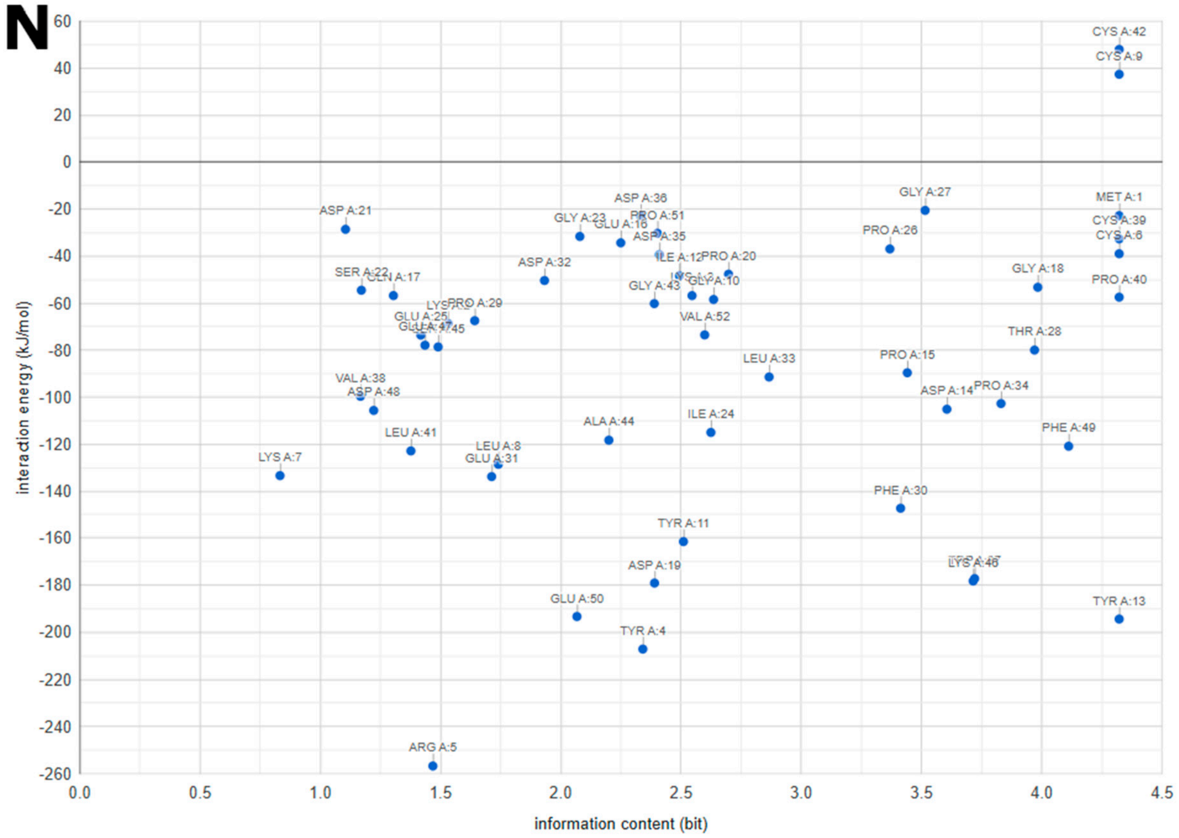
N
interaction energy (kJ/mol)
information content (bit)
60
40
20
0
-20
-40
-60
-80
-100
-120
-140
-160
-180
-200
-220
-240
-260
0.0
0.5
1.0
1.5
2.0
2.5
3.0
3.5
4.0
4.5
CYS A:42
CYS A:9
MET A:1
CYS A:39
CYS A:6
PRO A:40
ASP A:21
ASP A:36
GLY A:23
PRO A:51
GLU A:16
ASP A:35
GLY A:27
PRO A:26
ILE A:12
PRO A:20
ASP A:32
GLY A:18
SER A:22
GLY A:43
GLY A:10
PRO A:29
GLU A:25
VAL A:52
THR A:28
LEU A:33
PRO A:15
VAL A:38
ASP A:48
ASP A:14
PRO A:34
ILE A:24
ALA A:44
LEU A:41
PHE A:49
LEU A:8
GLU A:31
LYS A:7
PHE A:30
TYR A:11
LYS A:46
ASP A:19
GLU A:50
TYR A:13
TYR A:4
ARG A:5

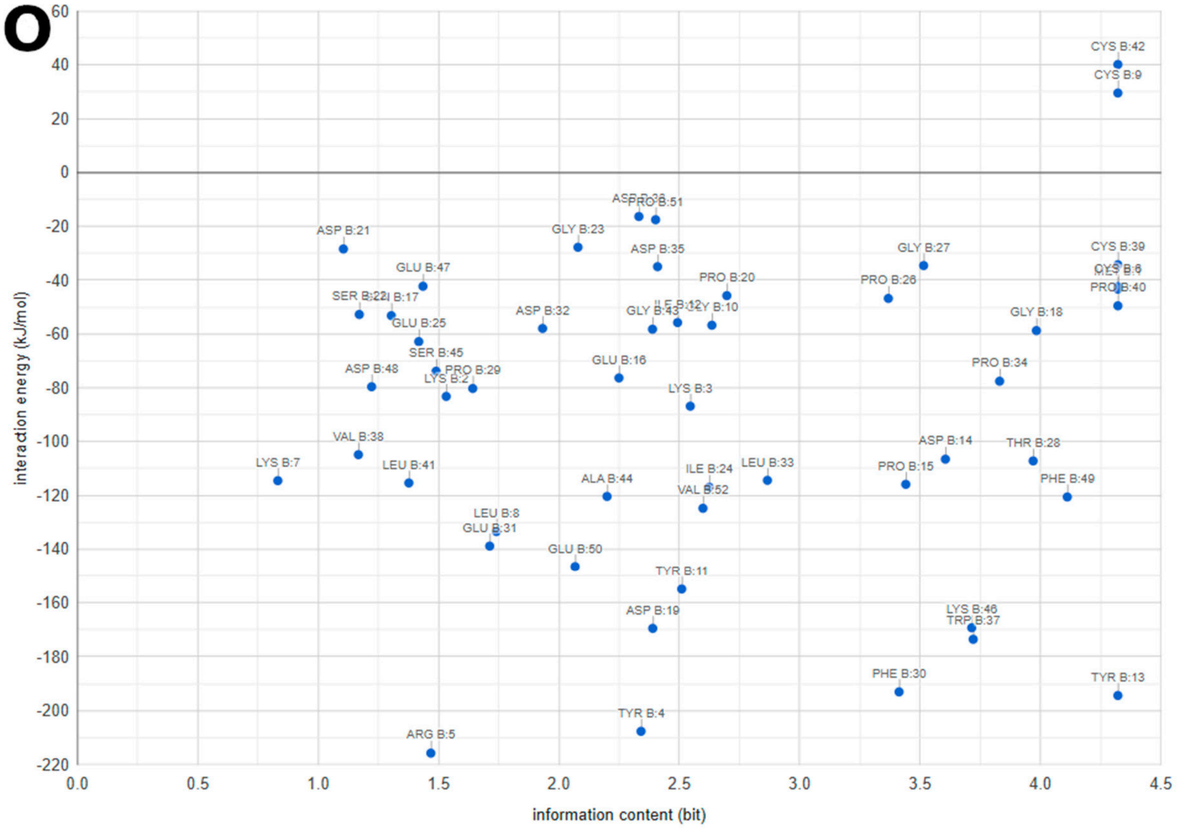

Figure S6. Total interaction energy (IE) versus information content (IC) scatter plot for the discussed rubredoxin structures as calculated at the INTAA web server. Universally conserved residues are clustered at the right edge (x=4.2), while the most favorable interaction energy is at the bottom of the y-axis. **A**. Fe *Pf* Rd sg19. **B**. Fe *Cpa* Rd(1FHH). **C**. Zn *Cpsy* Rd. **D**. Fe *Pg* Rd_subunit A, **E**. Fe *Pg* Rd_subunit B, **F.** Fe *Pg*R d_subunit C, **G**. Fe *Pg* Rd_subunit D, **H**. Fe *Pg* Rd_subunit E, **I**. Fe *Pg* Rd_subunit F, **J**. Fe *Pg* Rd_subunit G, **K**. Fe *Py* Rd_subunit A, **L**. Fe *Py* Rd_subunit B, **M**. Fe *Py* Rd_subunit C, **N**. Zn *Tm* Rd_subunit A, **O**. Zn *Tm* Rd_subunit B.
